# Lentiviral vector-mediated overexpression of mutant ataxin-7 recapitulates SCA7 pathology and promotes accumulation of the FUS/TLS and MBNL1 RNA-binding proteins

**DOI:** 10.1186/s13024-016-0123-2

**Published:** 2016-07-28

**Authors:** Sandro Alves, Thibaut Marais, Maria-Grazia Biferi, Denis Furling, Martina Marinello, Khalid El Hachimi, Nathalie Cartier, Merle Ruberg, Giovanni Stevanin, Alexis Brice, Martine Barkats, Annie Sittler

**Affiliations:** 1 INSERM U 1127, CNRS UMR 7225, Sorbonne Universités UPMC, Univ Paris 06 UMR_S 1127, ICM (Brain and Spine Institute) Pitié-Salpêtrière Hospital, 75013 Paris, France; 2 CNRS FRE3617, Center for Research in Myology, Sorbonne Universités UPMC Univ Paris 06, INSERM UMRS974, Institut de Myologie, G-H Pitié-Salpêtrière, 75013 Paris, France; 3 EPHE Ecole Pratique des Hautes Etudes, Laboratoire de Neurogénétique, PSL Universités, 75013 Paris, France; 4 INSERM UMR1169, MIRCen, 92265 Fontenay aux Roses, France; 5 Département de Génétique et Cytogénétique, AP-HP, G-H Pitié-Salpêtrière, 47 Bd de l’Hôpital, 75013 Paris, France

**Keywords:** Lentiviral vector, SCA7 mouse model, Ataxia, SCA7 patients, RNA binding-proteins

## Abstract

**Background:**

We used lentiviral vectors (LVs) to generate a new SCA7 animal model overexpressing a truncated mutant ataxin-7 (MUT ATXN7) fragment in the mouse cerebellum, in order to characterize the specific neuropathological and behavioral consequences of the genetic defect in this brain structure.

**Results:**

LV-mediated overexpression of MUT ATXN7 into the cerebellum of C57/BL6 adult mice induced neuropathological features similar to that observed in patients, such as intranuclear aggregates in Purkinje cells (PC), loss of synaptic markers, neuroinflammation, and neuronal death. No neuropathological changes were observed when truncated wild-type ataxin-7 (WT ATXN7) was injected. Interestingly, the local delivery of LV-expressing mutant ataxin-7 (LV-MUT-ATXN7) into the cerebellum of wild-type mice also mediated the development of an ataxic phenotype at 8 to 12 weeks post-injection. Importantly, our data revealed abnormal levels of the FUS/TLS, MBNL1, and TDP-43 RNA-binding proteins in the cerebellum of the LV-MUT-ATXN7 injected mice. MUT ATXN7 overexpression induced an increase in the levels of the pathological phosphorylated TDP-43, and a decrease in the levels of soluble FUS/TLS, with both proteins accumulating within ATXN7-positive intranuclear inclusions. MBNL1 also co-aggregated with MUT ATXN7 in most PC nuclear inclusions. Interestingly, no MBNL2 aggregation was observed in cerebellar MUT ATXN7 aggregates. Immunohistochemical studies in postmortem tissue from SCA7 patients and SCA7 knock-in mice confirmed SCA7-induced nuclear accumulation of FUS/TLS and MBNL1, strongly suggesting that these proteins play a physiopathological role in SCA7.

**Conclusions:**

This study validates a novel SCA7 mouse model based on lentiviral vectors, in which strong and sustained expression of MUT ATXN7 in the cerebellum was found sufficient to generate motor defects.

**Electronic supplementary material:**

The online version of this article (doi:10.1186/s13024-016-0123-2) contains supplementary material, which is available to authorized users.

## Background

Spinocerebellar ataxia type 7 (SCA7) is an inherited autosomal dominant neurodegenerative disorder. Patients present with cerebellar ataxia due to moderate to severe neuronal loss and gliosis in the cerebellum, especially Purkinje cells, inferior olivary, dentate nucleus and pontine nuclei, and to a lesser extent in the *globus pallidus*, *substantia nigra* and *red nucleus*. They also present with visual impairment due to degeneration of cone and rod photoreceptors [1–3]. SCA7 is caused by an unstable CAG repeat expansion in the coding region of the *SCA7* gene conferring a toxic gain of function to the ataxin-7 (ATXN7) protein which accumulates aberrantly in neurons, a mechanism also involved in a family of eight other inherited neurodegenerative polyglutamine (PolyQ) diseases, including Huntington’s disease (HD), spinobulbar muscular atrophy (SBMA), dentatorubral pallidoluysian atrophy (DRPLA), spinocerebellar ataxia (SCA) types 1, 2, 3, 6 and 17 [4]. ATXN7 is ubiquitously expressed in the brain and is a component of the highly conserved transcriptional coactivator Spt/Ada/Gcn5 acetylase (SAGA) chromatin remodelling complex with histone acetyltransferase activity and deubiquitinase activity [5]. It has been shown that the ubiquitin protease activity of SAGA is important for the expression of tissue-specific and developmental genes [5]. Recently, it was shown that SAGA acetylates the promoters and deubiquitinates the transcribed regions of all expressed genes [6]. ATXN7 has been described to be cleaved by caspase-7 at two sites [7], generating N-terminal fragments containing the polyQ tract, resulting in MUT ATXN7 fragments that accumulate in the nucleus. Indeed, a ∼ 55 kDa ATXN7 amino-terminal fragment was previously identified in SCA7 transgenic mice and in SCA7 patients [8]. Interestingly, it has been reported that post-translational modifications at lysine 257, adjacent to the caspase-7 mediated cleavage site of ATXN7 at position 266, mitigate fragment accumulation in vitro and in vivo, thus regulating SCA7 toxicity [9, 10]. ATXN7 expanded polyQ stretches result in conformational modifications, finally leading to the formation of insoluble aggregates, hallmarks of SCA7 [11]. The exact mechanism by which polyQ aggregates mediate toxicity is still debated, but one strong hypothesis is the fact that they may be prone to trap multiple binding partners such as transcription factors, important to the maintenance of cell homeostasis, that will in turn be progressively depleted [3], or RNA-binding proteins (RBPs), leading to dysregulation of alternative splicing of target mRNAs [12, 13]. The generation of murine genetic models that closely recapitulate the human neuropathology are extremely valuable for the dissection of disease mechanisms and evaluation of therapeutic strategies. In the case of SCA7, the cloning of the *SCA7* gene allowed the creation of transgenic and knock-in mouse models, in which cerebellar neuronal dysfunction and progressive retinal degeneration were directly associated to the accumulation of mutant ATXN7 [8, 14–16], despite poor neuronal degeneration [8, 15]. Alternatively, local overexpression of mutant proteins using viral vectors has been a successful strategy to model polyQ pathologies of the central nervous system (CNS), such as HD [17] and SCA3 [18], generating robust in vivo genetic models leading to neuronal degeneration in well-defined brain regions.

Here, we generated an in vivo model of SCA7 by overexpressing truncated MUT ATXN7 in the mouse cerebellum using a locally injected lentiviral vector (LV). The truncated construct we used corresponds approximately to the caspase-7 cleavage fragment [7]. The SCA7-LV mice developed an ataxic phenotype and this model further allowed investigating whether specific RBPs could be involved in the pathogenesis of SCA7. We initially focused on the RBP *Fused in sarcoma* (FUS/TLS), found to be mutated in familial amyotrophic lateral sclerosis (ALS) [19], since it was shown to be a major component of nuclear aggregates in several polyQ disorders, such as HD, SCA1 and SCA3 [20, 21]. We next looked for the transactive response DNA binding protein 43-kDa (TDP-43), pathologically associated to ALS and frontotemporal lobar degeneration with ubiquitinated inclusions (FTLD-U) [22] that was also shown to be sequestered in polyQ aggregates [23]. Finally, we investigated in this SCA7-LV model, the cellular localization and expression of two evolutionarily conserved RBPs that constitute part of the muscleblind-like protein family (MBNL1 and MBNL2) and are expressed in a wide variety of adult tissues including brain, heart and skeletal muscle [24]. MBNL1 and MBNL2 associate and bind to expanded CUG and CAG repeats, which accumulate as discrete nuclear foci in both DM1 and DM2 (myotonic dystrophy type 1 and type 2) [24–26], therefore suggesting their implication in these disorders.

We therefore investigated in the LV-based model of SCA7 the impact of MUT ATXN7 accumulation over the expression of the RBPs referred above. This was complemented with immunohistochemical and biochemical analyses performed in the cerebellum of a SCA7 knock-in mouse model [16] and in cerebella from SCA7 affected patients. In summary, we set up a new LV-based model of SCA7, alternative to transgenesis, in which specific RBPs were found to be accumulated, suggesting their role in SCA7 pathogenesis.

## Results

### Lentiviral-mediated MUT ATXN7 overexpression in the mouse cerebellum triggers time-dependent aggregation

In SCA7 KI mice, mutant ATXN7 accumulates robustly in the cerebellum [16, 27]. Since human SCA7 pathology strongly affects the cerebellum (reviewed in [28]), we created a SCA7 LV-based model in which LV encoding human ATXN7, driven by the PGK-1 (phosphoglycerate kinase 1) promoter, is injected into the mouse cerebellum.

We first checked that intracerebellar injection of GFP-expressing LVs allowed a sustained expression of the transgene over a large region (~3 mm), almost exclusively in neurons, as demonstrated herein by GFP-immunohistochemical analysis at 12 weeks post-injection (Additional file 1: Fig. S1).

**Fig. 1:**
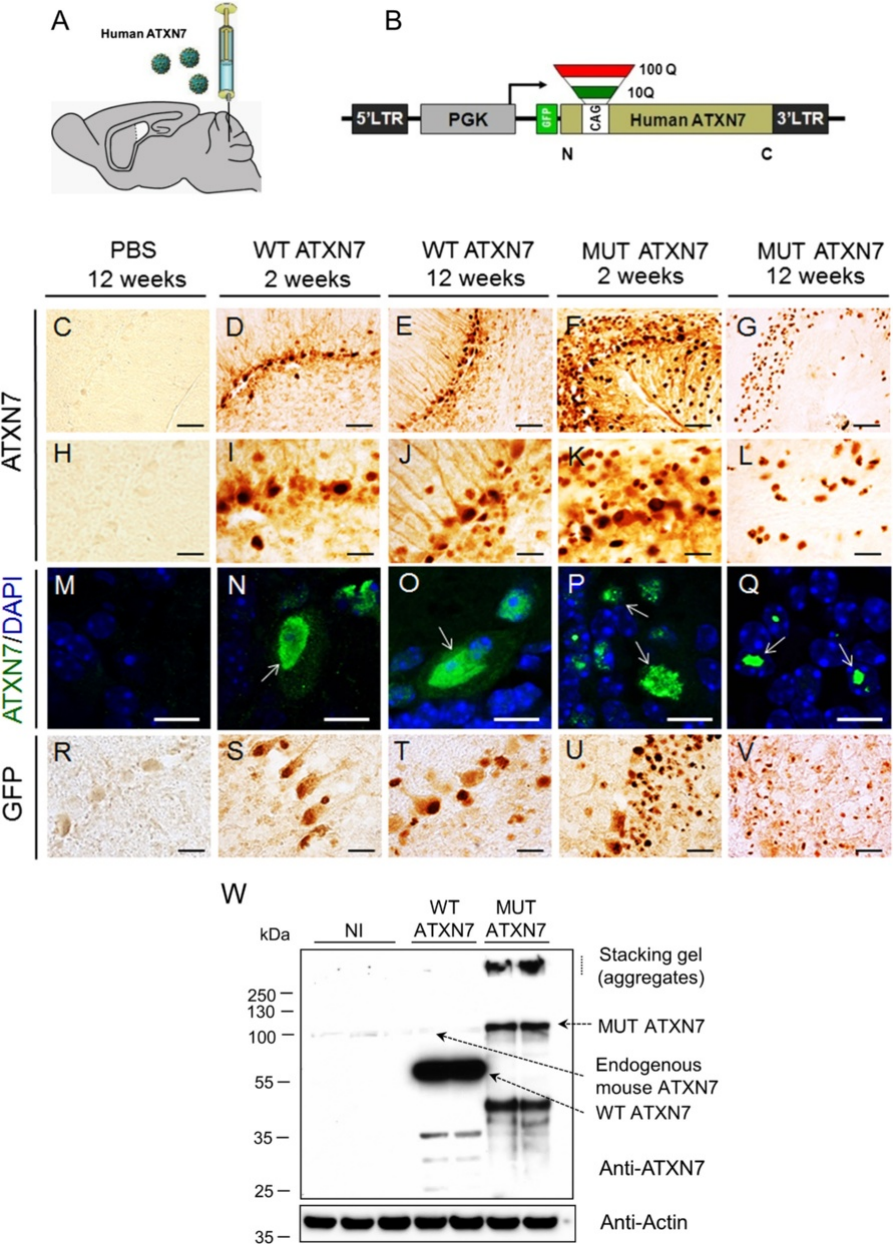
Lentiviral-mediated overexpression of truncated human WT ATXN7 and MUT ATXN7 in the mouse cerebellum. **a** Protocol: lentiviral vectors (LV) encoding truncated human WT ATXN7 or MUT ATXN7 were injected sterotactically into the mouse cerebellum. The animals were sacrificed at 2 and 12 weeks post-injection. **b** Schematic representation of the LV encoding truncated (amino acids 1–230) wild-type (WT ATXN7; 10 CAG repeats) or mutant (MUT ATXN7; 100 CAG repeats) human ATXN7 (ATXN7) and a GFP tag, under the control of the phosphoglycerate kinase-1 (PGK-1) promoter. **c**–**l** and (**r**–**v**) Immunohistochemistry and (**m**–**q**) immunohistofluorescence in cerebellum. Truncated WT ATXN7 was mainly nuclear and diffuse in PCs at 2 (D, I and N) and 12 weeks post-injection (E, J and O) (arrows), whereas MUT ATXN7 was nuclear and diffuse at 2 weeks (F and K), with small intranuclear aggregates in PCs (arrows in P) (laser confocal microscopy: ATXN7 in green; DAPI stain, blue). MUT ATXN7 forms robust intranuclear inclusions in the GCL at 12 weeks (G, L and arrows in Q). Immunohistochemical labeling with anti-GFP showed similar results (R-V). No transgenic ATXN7 immunoreactivity was observed in mice injected with PBS (C, H and M). **w** Representative western-blot of cerebellar lysates: overexpression of truncated WT ATXN7 and MUT ATXN7, 2 weeks post-injection, probed with an anti-ATXN7 antibody that recognizes both endogenous mouse and human ATXN7. Non-injected cerebella (NI) were used as control: human WT ATXN7 expression was ~30-fold higher than endogenous mouse ATXN7 (analysis by optical densitometry). ATXN7-positive aggregates were retained in the stacking gel (SDS-insoluble fraction) and a robust smear of putative cleavage fragments was observed in MUT ATXN7 but not in WT ATXN7-injected animals. Bars: C–G: 50 μm; H–L: 20 μm; M–Q: 10 μm; R–V: 20 μm

Wild-type mice were therefore injected following similar stereotactical conditions with LVs expressing either the wild-type (LV-ATXN7-10Q, *n* = 9) or the mutant ATXN7 (LV-ATXN7-100Q, *n* = 11), driven by the PGK-1 (phosphoglycerate kinase-1) promoter (Fig. 1a, b). WT ATXN7 and MUT ATXN7 were detected in the cerebellum 2 weeks and 12 weeks after LV injection using an antibody against ATXN7, thus modelling an early or late stage of the SCA7 pathology. The intense anti-ATXN7 immunostaining revealed the production of a significant amount of both the wild-type and mutant human protein within the PC layer and the cells of the granule and molecular layers (Fig. 1d–g). The immunoreactivity of truncated WT ATXN7 was mainly nuclear and diffuse at 2 weeks (Fig. 1d, i and n) and 12 weeks post-injection (Fig. 1e, j and o). Similarly, MUT ATXN7 immunostaining was nuclear and relatively diffuse at 2 weeks post-injection (Fig. 1f and k), however the formation of early small aggregates was already evidenced by laser confocal microscopy (Fig. 1p). As expected, a robust nuclear and punctuate staining of MUT ATXN7 was observed at 12 weeks post-injection (Fig. 1g, l and q), corresponding to the nuclear ATXN7-positive inclusions, hallmarks of SCA7. No transgenic ATXN7 was observed in mice injected with PBS (Fig. 1c, h and m). Similar results were obtained using anti-GFP immunohistochemistry to distinguish the human from the murine endogenous ATXN7 (Fig. 1r–v).

Upon injection of the vectors in the mouse cerebellum, truncated human WT ATXN7 and MUT ATXN7 were also detected by western-blot on tissue biopsies at 2 weeks post-injection. The level of transgenic WT ATXN7 was ~30 times higher than endogenous murine ATXN7, indicating that the LVs strongly overexpress human ATXN7 in vivo. The amount of soluble transgenic MUT ATXN7 was lower, compared to WT ATXN7, probably due to the strong accumulation in the form of SDS-insoluble aggregates, observed in the stacking gel (Fig. 1w).

In regions expressing MUT ATXN7, misfolded ubiquitin-positive and p62 proteins co-localized with ATXN7 in ~93 and ~80 % of nuclear inclusions at 12 weeks post-injection, as described previously in a knock-in mouse model containing 266 glutamines [29]. In contrast, no ubiquitin or p62 accumulation was detected in regions injected with vectors encoding truncated WT ATXN7 (Additional file 1: Fig. S2).

### Lentiviral-based overexpression of MUT ATXN7 in the mouse cerebellum induces neuronal cell loss, disruption of axons and dendrites and synaptotoxicity

**Fig. 2:**
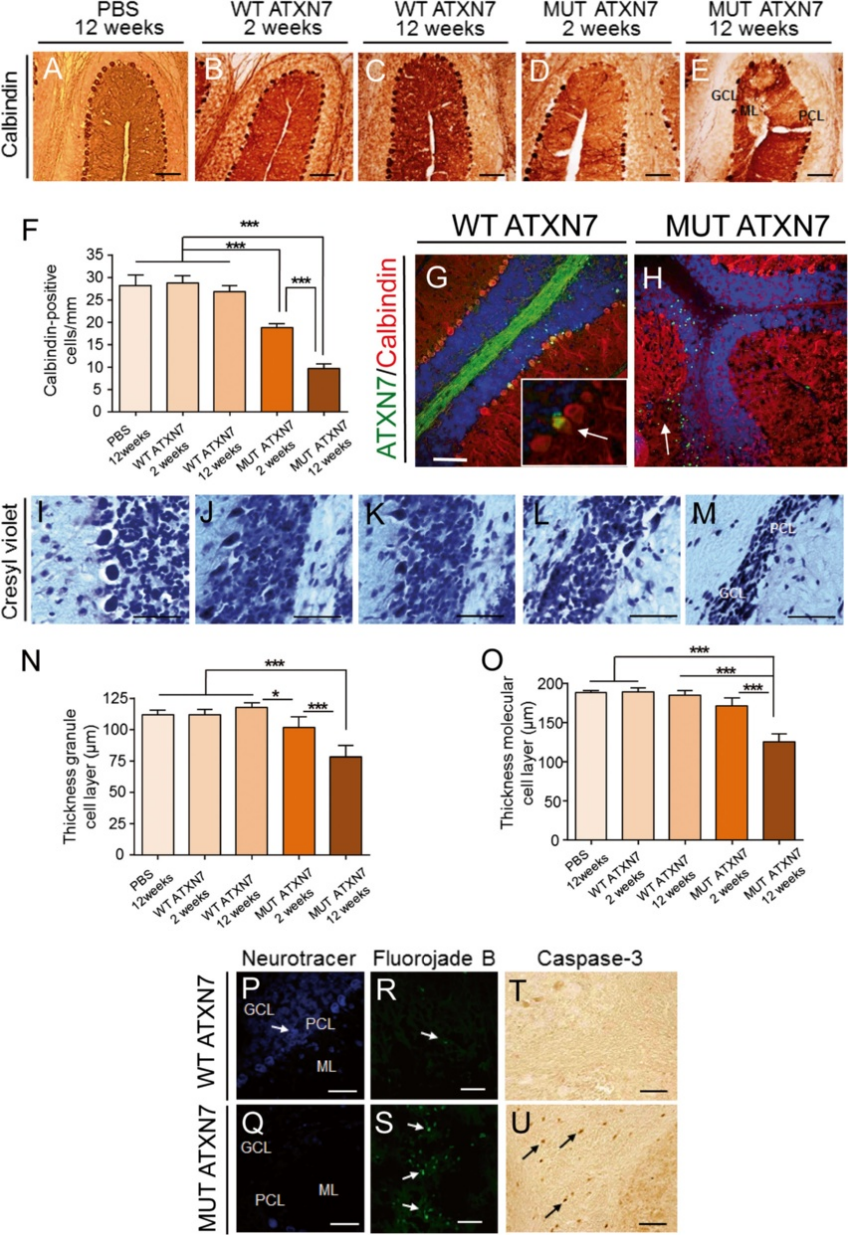
Lentiviral-based overexpression of MUT ATXN7 induces cell loss and degeneration in the mouse cerebellum. **a**–**e** Calbindin immunostaining revealed loss of Purkinje cells (PC) in mice injected with an LV-MUT-ATXN7, 2 and 12 weeks post-injection (D and E); in mice injected with PBS or an LV-WT-ATXN7 no cell loss was observed at either 2 weeks or 12 weeks post-injection (A, B and C). **f** Quantification: in transduced regions, the number of calbindin-positive PCs decreased ~19 %, 2 weeks post-injection and ~64 % 12 weeks post-injection in MUT ATXN7 transduced regions, whereas injection with PBS or overexpression of WT ATXN7 did not affect calbindin-positive PCs. Values are expressed as mean ± standard deviation (SD). **g** and **h** Laser confocal microscopy showing the preservation of calbindin-positive PCs (red) in regions transduced with WT ATXN7 displaying diffuse immunoreactivity (green) (arrow in G); PCs (red) were depleted in areas transduced with LV-MUT ATXN7, attested by the presence of ATXN7-positive aggregates (green). **i**–**m** Cresyl violet staining showed preservation of PCs and GCL in mice injected with PBS or LV-WT-ATXN7, whereas loss of PCs and shrinkage of the GCL (L and M) were observed in mice injected with MUT ATXN7, at 2 and 12 weeks post-injection. **n**–**o** Quantification of GCL and ML thickness: overexpression of MUT ATXN7 induced a shrinkage of the GCL (~31 %) (M and N) and ML (~33 %) (M and O) near the injection site, 12 weeks post-injection. PBS injection or overexpression of WT ATXN7 did not affect PCs, the ML or the GCL thickness (I, J, K, N and O). Statistical analyses of calbindin immunostaining and cresyl violet staining were performed by one-way ANOVA followed by a *post-hoc* Fisher’s test. Values are expressed as mean ± standard deviation (SD). **p**–**q** Neurotracer staining is decreased in PCs and GCL (Q) in mice injected with LV-MUT-ATXN7 compared to mice injected with LV-WT-ATXN7 (P). **r**–**s** Fluoro-Jade B staining shows increased number of degenerating neurons in mice injected with LV-MUT-ATXN7 (S) compared to mice overexpressing WT ATXN7 (R). **t**–**u** The number of caspase-3-positive cells increased in cerebellar regions injected with LV-MUT-ATXN7 (U). Bars: A–E: 100 μm; G and H: 100 μm; I–M: 50 μm; P–Q: 20 μm; R–S: 50 μm; T–U: 20 μm

We then investigated whether overexpression of MUT ATXN7 could trigger dysfunction followed by neurodegeneration, by carrying out immunohistochemical analysis for the calbindin-D28K protein, a specific marker for PCs (Fig. 2a–e). In MUT ATXN7-injected mice, the immunostaining analysis performed at 2 and 12 weeks post-injection showed either moderate (21.33 ± 2.43 cells/mm) (Fig. 2d and f) or robust loss of PCs (9.63 ± 1.13 cells/mm) (Fig. 2e and f), respectively. In contrast, no cell loss was observed 2 weeks (28.12 ± 2.56 cells/mm) (Fig. 2b and f) or 12 weeks post-injection (26.4 ± 0.48 cells/mm) (Fig. 2e and f) in mice that received LV-WT-ATXN7, compared to mice injected with PBS at 12 weeks (Fig. 2a and f). Double staining for WT ATXN7 and calbindin revealed that a high proportion of PCs expressed the wild-type protein at 12 weeks after LV injection, without inducing any toxicity (Fig. 2g). PC cells also expressed MUT ATXN7 early after LV-injection (2 weeks) (Additional file 1: Fig. S3) but not at 12 weeks post-injection most probably due to massive PC loss (Fig. 2h).

**Fig. 3:**
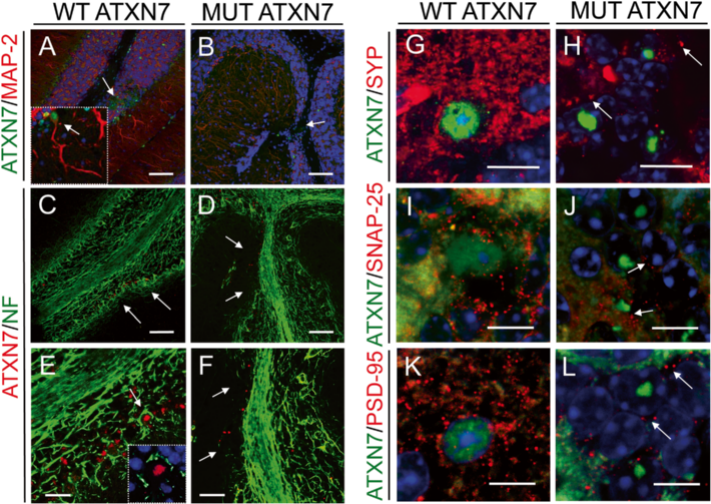
MUT ATXN7 disrupts cerebellar axonal and dendritic structure and causes synaptotoxicity. Laser confocal microscopy showing loss of microtubule associated protein 2 (MAP-2) immunoreactivity (red) in the molecular layer, in cerebellar regions injected with LV-MUT-ATXN7 (green) (**b**); in regions transduced with WT ATXN7, MAP-2-positive arborizations were preserved (**a**); high magnification inset: WT ATXN7 immunoreactivity (green) in the nucleus of PCs with standard MAP-2 immunoreactivity (red) (A). Loss of NF70-kDa (NF: neurofilament) immunoreactivity (green) around the injection site was observed when MUT ATXN7 (red) was overexpressed in cerebellum (**d**) and (**f**). The standard pattern of neurofilaments (NF) immunoreactivity around PCs and in the GCL was observed in regions (arrows) transduced with WT ATXN7 (**c**) and (**e**); high magnification inset: WT ATXN7 immunoreactivity (red) in PC nuclei with standard NF-immunoreactivity (green) around PCs. Loss of synaptophysin (SYP) (red) (**h**), synaptosomal-associated protein 25 (SNAP-25) (red) (**j**) and postsynaptic density protein 95 (PSD-95) (red) (**l**) immunoreactivity in MUT ATXN7-transduced regions (green) (GCL); standard dot-like SYP, SNAP-25 and PSD-95 immunoreactivity was observed in WT ATXN7-expressing areas (PCs and in GCL) ((**g**), (**i**) and (**k**), respectively). Bars: A–D: 100 μm; E–F: 50 μm; G–L: 10 μm

In addition, cresyl violet stained sections (Fig. 2i–m) showed the presence of atrophic cell nuclei (Fig. 2m) and shrinkage of both the granule (GCL) (~31 %) and molecular layers (ML) (~33 %) around the injection site, only at 12 weeks post-injection in mice injected with LV-MUT-ATXN7 (Fig. 2n and o, respectively). Finally, neurotracer (Fig. 2p and q) and Fluorojade-B (Fig. 2r and s) dyes were used to stain degenerating cells, and caspase-3 (Fig. 3t and u), an apoptosis-related cysteine peptidase, was used to label apoptotic cells: in cerebellar regions injected with LV expressing truncated WT ATXN7 no degenerating and/or apoptotic neurons were observed, whereas a large number of degenerating cells were detected in regions injected with LV expressing truncated MUT ATXN7 (Fig. 3s and u, arrows).

Next, we examined the impact of both WT ATXN7 and MUT ATXN7 overexpression on two major neuron-specific cytoskeletal proteins: the microtubule associated protein 2 (MAP-2), a marker for dendritic processes, and the neurofilament (NF) 70-kDa protein, that provide support for normal axonal radial growth. Double staining of cerebellar sections from LV-MUT-ATXN7 injected mice with antibodies against ATXN7 and either MAP-2 (Fig. 3b) or NF (Fig. 3d and f), showed a weak immunoreactivity at the site of LV injection (where transgene expression levels are the highest) very likely due to local cell loss and subsequent disruption of both axonal and dendritic networks. As expected, no alteration in the distribution of MAP-2 (Fig. 3a) and NF (Fig. 3c and e) was observed after LV-WT-ATXN7 injection.

Because synaptotoxicity has not yet been examined in rodent models of SCA7, we investigated whether this pathological feature would be detected in our LV-based SCA7 mouse model and correlated with MUT ATXN7 accumulation. For this purpose, we carried out immunohistochemical staining for the synaptophysin (SYP), synaptosomal-associated protein 25 (SNAP-25) and post-synaptic density protein 95 (PSD-95) proteins, which are all associated to the synaptic compartment. The immunoreactivity of SYP, SNAP-25 and PSD-95 was clearly decreased in the LV-MUT-ATXN7 injected cerebellar regions (Fig. 3h, j and l, respectively), as compared to those injected with the LV-WT-ATXN7 (Fig. 3g, i and k, respectively), which revealed a strong immunoreactivity of these synaptic markers. Altogether, these results showed that LV-based delivery of MUT ATXN7 in wild-type mice induced a loss of PCs as well as synaptotoxicity.

### Cerebellar overexpression of MUT ATXN7 induces up-regulation of inflammation markers

**Fig. 4:**
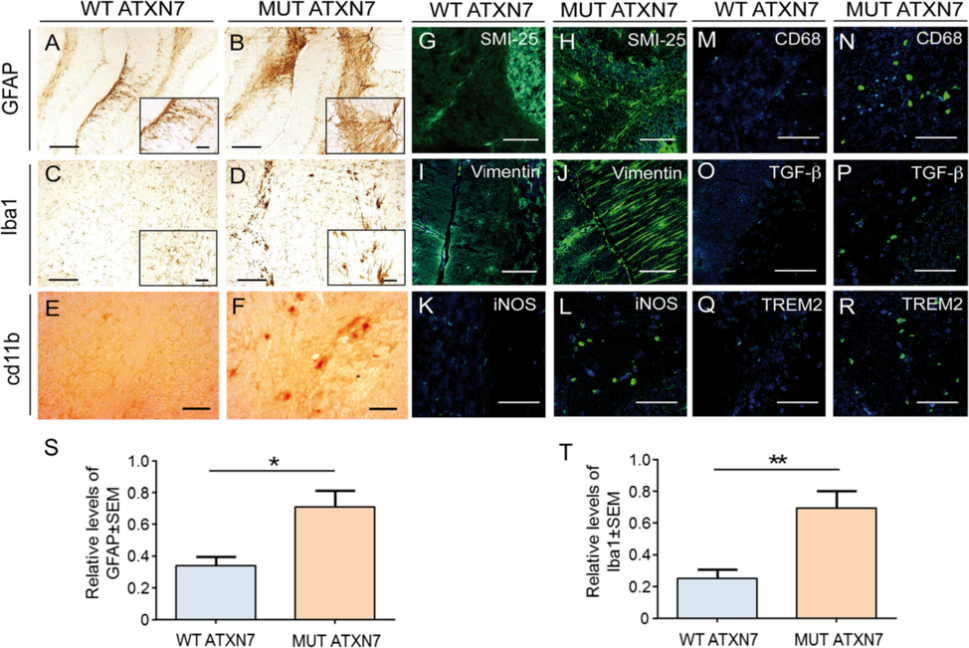
Cerebellar overexpression of MUT ATXN7 induces immunoreactivity of markers of inflammation. **a**–**f** Cerebellum injected with LV-MUT-ATXN7 shows increased GFAP (B), Iba1 (microglia) (D) and cd11b (F) immunoreactivity compared to a cerebellum injected with WT ATXN7 (A, C and E, respectively). **g**–**r** Laser confocal microscopy shows increased astrocytic immunoreactivity (SMI-25 staining; green) in cerebellar regions transduced with MUT ATXN7 (H) compared to control regions injected with WT ATXN7 (G); Anti-vimentin staining (green) shows increased immunoreactivity of Bergmann glial cells in the cerebellar ML transduced with MUT ATXN7 (J) relatively to the ML transduced with WT ATXN7 (I). The increased immunoreactivity of the microglial markers NOS2 (L), CD68 (N), TGF-ß (P) and TREM2 (R) (in green) is observed in cerebellar regions overexpressing MUT ATXN7, compared to regions overexpressing WT ATXN7 (K, M, O and Q, respectively). **s** and **t** Quantification showing increased GFAP-positive immunoreactivity (S) and Iba1-positive immunoreactivity in the cerebellum of mice injected with LV encoding MUT ATXN7 (*n* = 4), relatively to WT ATXN7-injected mice (*n* = 4); Student’s *T* test. Values are expressed as mean ± standard error of the mean (SEM). Bars: A–D: 200 μm; E and F: 50 μm; G–J: 50 μm; K–R: 20 μm

We next studied the consequences of human ATXN7 overexpression over markers accompanying inflammation. Our results demonstrated that, compared to LV-WT-ATXN7 injected mice, the animals overexpressing the LV-MUT-ATXN7 showed a local up-regulation of markers associated to inflammation, such as the Glial Fibrillary Acidic Protein (GFAP) astrocytic marker (Fig. 4b; quantification in S), the microglia/macrophage-specific calcium-binding protein ionized calcium binding adaptor molecule 1 (Iba1) (Fig. 4d; quantification in T) and the cd11b pan-macrophage marker (Fig. 4f). These data were supported by additional immunofluorescence analyses using another anti-GFAP antibody (SMI-25) and vimentin that revealed intense immunoreactivity in astrocytes and Bergmann glial cells, respectively, in regions injected with LV-MUT-ATXN7, compared to regions injected with LV-WT-ATXN7 (Fig. 4g–j). In addition, specific microglial markers, such as the nitric oxide synthase (iNOS), CD68, the Transforming growth factor Beta (TGF-ß) and the Triggering receptor expressed on myeloid cells 2 (TREM2) (Fig. 4k–r) were shown to be-upregulated in regions injected with LV-MUT-ATXN7, but not in the cerebella of mice injected with LV-WT ATXN7.

### Lentiviral-mediated overexpression of MUT ATXN7 in the cerebellum induces ataxic behavioral deficits in mice

**Fig. 5:**
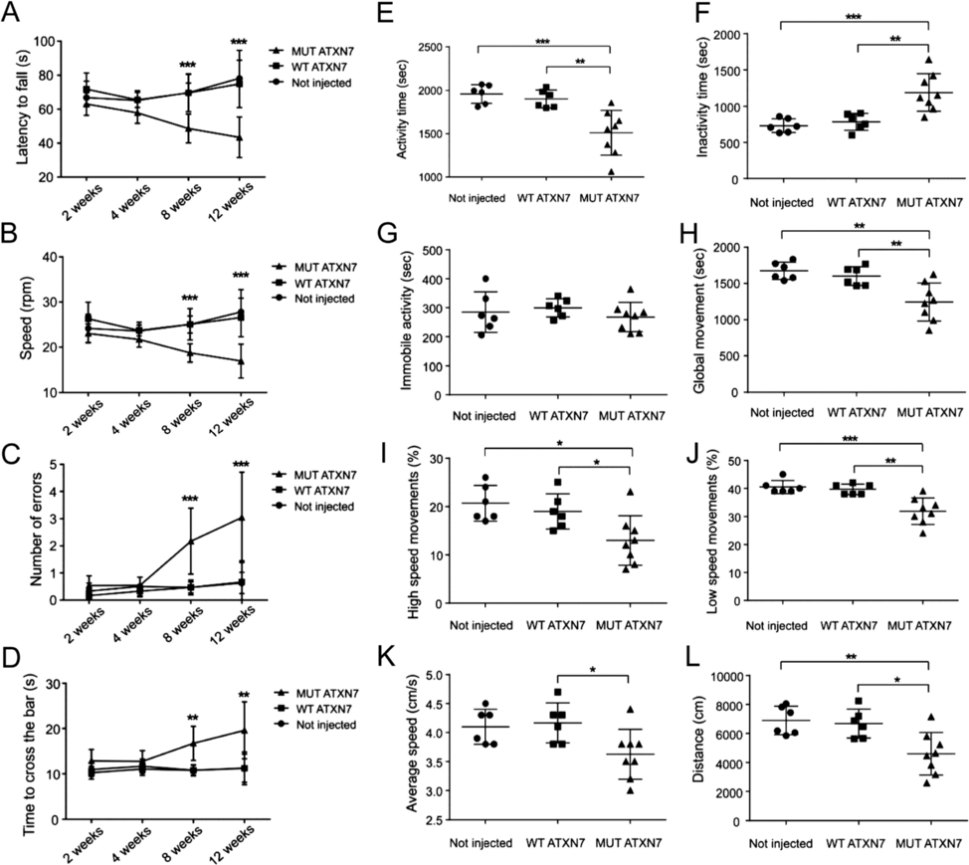
Lentiviral-mediated MUT ATXN7 overexpression in the mouse cerebellum induces locomotor abnormalities. Performance on the accelerating rotarod showed reduced motor coordination at 8 and 12 weeks, but not at 2 or 4 weeks post-injection in mice injected with LV-MUT-ATXN7 (**a**-latency to fall; **b**-speed at falls), relative to non-injected mice or mice injected with LV-WT-ATXN7. The locotronic test shows an increased number of errors (**c**) and time to cross the bar (**d**) in mice injected with LV-MUT-ATXN7, relatively to non-injected mice or mice receiving LV-WT-ATXN7, at 8 and 12 weeks post-injection. Measure of actimetry: total active time decreased (~20 %) (**e**) and inactive time increased (**f**), but no statistically significant changes in total immobility (**g**) were observed in mice injected with MUT ATXN7. These mice also displayed reduced global movements (~22 %) (**h**), low (~20 %) (**j**) and high speed movements (~31.5 %) (**i**), average speed (~12 %) (**k**) and total distance traveled (~31 %) (**l**) during 45 min recording, compared to non-injected mice or mice injected with LV-WT ATXN7. The performances of mice injected with LV-WT-ATXN7 or non-injected mice were similar in all tests. Statistical analysis for actimetry assessment was performed using one-way ANOVA followed by a *post-hoc* Fisher’s test. Statistical analysis for behavior assessment in the rotarod and locotronic tests was performed using Two-way ANOVA followed by a post-hoc Fisher’s test. Number of animals; *n* = 6 for non-injected, *n* = 6 for WT ATXN7 and *n* = 8 for MUT ATXN7 injected mice.

To evaluate whether the neuropathological changes induced by MUT ATXN7 overexpression could be translated into behavioral dysfunction, the animals underwent two tests of balance and motor coordination, the rotarod and the locotronic motor tests, that are known to be sensitive to cerebellar dysfunction, at early (2 and 4 weeks) and late (8 and 12 weeks) disease stages. The spontaneous activity was also monitored using an automated activity cage (actimeter) at 12 weeks after injection (Fig. 5).

At an early disease stage (2 or 4 weeks after injection), no statistically significant difference was found in the rotarod test for the latency to fall (LF) and the speed when we compared the LV-MUT-ATXN7 mice and either the non-injected or the LV-WT-ATXN7 injected mice (Fig. 5a and b). At late stages (8 and 12 weeks post-injection), the overexpression of truncated MUT ATXN7 in the cerebellum induced a severe loss of motor coordination, with a statistically significant difference for the LF and the speed between the LV-MUT-ATXN7 injected group (LF: 48.7 ± 9.2 s; speed: 18,7 ± 2.04 rpm; *n* = 8) and either the non-injected mice (LF, 69,75 ± 5,71 s; speed, 25.03 ± 1.88 rpm; *n* = 6) or the LV-WT-ATXN7 injected mice (LF = 69,61 ± 11.03 s; speed = 25.06 ± 3.48 rpm; *n* = 6) at 8 weeks after injection. Similar data were observed, at 12 weeks after injection, with a statistically significant difference for the LF and the speed between the LV-MUT-ATXN7 injected group (LF: 43.5 ± 12 s; speed: 17 ± 3.7 rpm; *n* = 8) and either the non-injected mice (LF: 78.2 ± 11 s; speed, 27.8 ± 5 rpm; *n* = 6) or the LV-WT-ATXN7 injected mice (LF = 74.5 ± 13.9 s; speed = 26.56 ± 4.3 rpm).

The locomotor behavior of the LV-MUT-ATXN7 injected animals was also shown to be severely impaired in the locotronic test of fine paw motor coordination at 8 and 12 weeks post-injection. No statistically significant difference was found at 2 and 4 weeks post-injection between mice injected with the LV-MUT-ATXN7 and either non-injected or LV-MUT-ATXN7-injected mice, whereas the number of errors (NE) and the time spent to cross the bars (TCB) were robustly increased at a later disease stage (8 and 12 weeks post-injection) in mice overexpressing the truncated MUT ATXN7 (Fig. 5c and d).

Statistically significant differences were found at 8 weeks post-injection between the LV-MUT-ATXN7 injected group (Number of errors NE = 2.2 ± 1.2; TCB = 17.63 ± 3.86 s; *n* = 8) and the non-injected (NE = 0.47 ± 0.21; TCB = 10.8 ± 1.08 s; *n* = 6) or the LV-WT-ATXN7 injected mice (NE = 0.47 ± 0.27; TCB = 10.1 ± 2.03 s; *n* = 6). Similar results were observed at 12 weeks post-injection between the LV-MUT-ATXN7 injected mice (NE = 3.1 ± 1.7; TCB = 21.52 ± 7.76 s; *n* = 8) and the non-injected (NE = 0.63 ± 0.39; TCB = 10.6 ± 1.62 s; *n* = 6) or the LV-WT-ATXN7injected mice (NE = 0.67 ± 0.79; TCB = 10.20 ± 2.34 s; *n* = 6). Although a tendency was observed for an age-related aggravation of the phenotype between 8 and 12 weeks after LV-MUT-ATXN7 injection, the difference in performance between 8 and 12 weeks did not reach statistical significance (Fig. 5c and d). No statistically significant difference was found between non-injected mice and WT ATXN7 overexpressing mice at any time-point of the study (Fig. 5c and d).

Finally, the behavioral effects of MUT ATXN7 overexpression were analyzed by monitoring spontaneous motor activity in an actimeter during 45 min at 12 weeks post-injection (Fig. 5e–l). Again, the animals injected with LV-MUT-ATXN7 differed significantly from LV-WT-ATXN7-injected or non-injected mice, with an overall decrease of activity. Statistically significant differences were observed between LV-MUT-ATXN7 and either LV-WT-ATXN7 injected or non-injected mice in the total activity time (1510 ± 259 s, 1901 ± 103 s and 1958 ± 109 s, respectively) (~20 % of decrease), global movements (1243 ± 262 s, 1602 ± 130 s and 1673 ± 120 s, respectively) (~22 % of decrease), the percentage of low (31.9 ± 4.7, 39.67 ± 1.86 and 40.5 ± 2.35 %, respectively) (~20 %) and high speed (13 ± 5.13, 19 ± 3.63 and 20.67 ± 3.67 %, respectively) (~31.5 %) movements, the average speed (3.63 ± 0.43 cm/s, 4.17 ± 0.34 cm/s and 4.1 ± 0.3 cm/s, respectively) (~12 %) and the total distance traveled (4598 ± 1466 cm, 6682 ± 994 cm and 6898 ± 979 cm, respectively) (~31 %). No statistically significant difference was found between LV-WT-ATXN7 injected and non-injected mice (Fig. 5e–l).

### Nuclear MUT ATXN7 inclusions sequester FUS/TLS and accumulate phosphorylated TDP-43

**Fig. 6:**
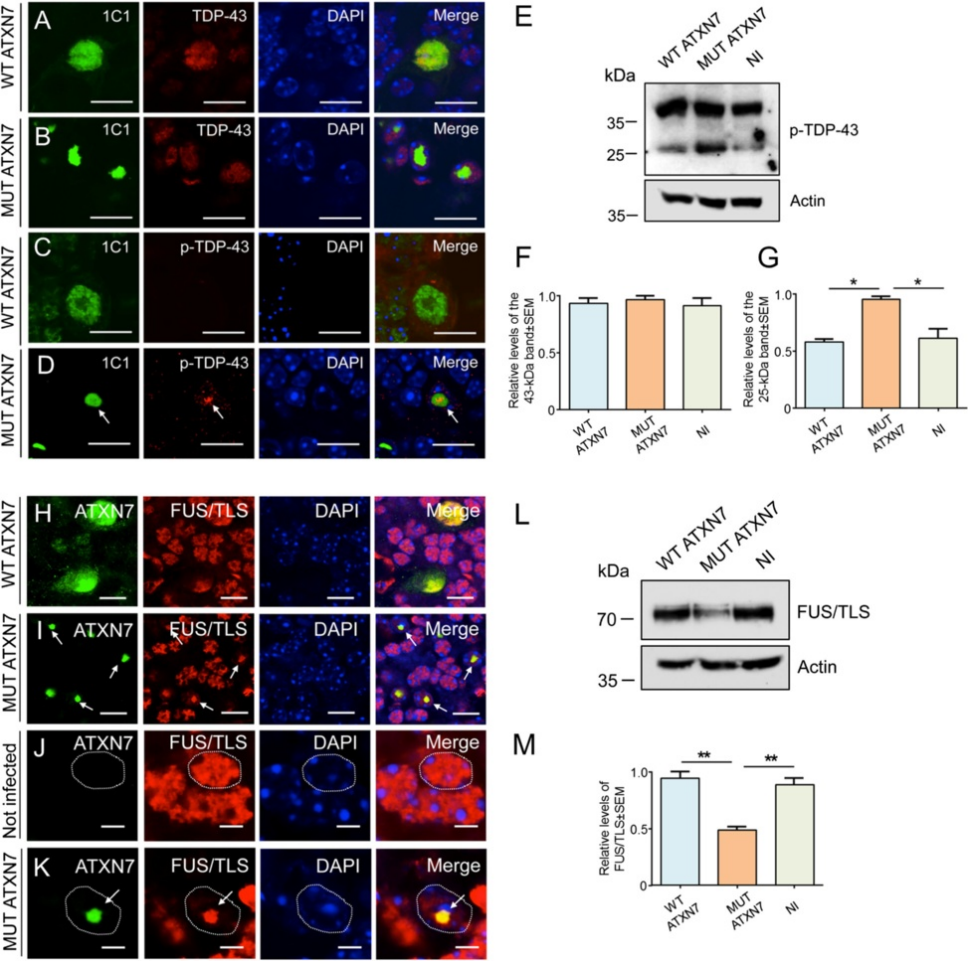
Phosphorylated TDP-43 and FUS/TLS accumulate in the cerebellum of the LV-mouse model of SCA7. Laser confocal microscopy showing that murine TDP-43 (red) remains diffuse in a Purkinje cell transduced with WT ATXN7 (green) at 12 weeks post-injection (**a**). Murine TDP-43 (red) does not co-aggregate in MUT ATXN7-positive inclusions (green) at 12 weeks post-injection (**b**). Partial co-localization of MUT ATXN7 (green) and phosphorylated TDP-43 (red) (~12 %) was observed in GCL (**d**); nuclear WT ATXN7 (green) does not co-localize with phosphorylated TDP-43 (**c**) in PCs (*n* = 5). **e** Representative western-blot of cerebellar lysates shows a ~1.6-fold increased level of a 25-kDa band of phosphorylated-TDP-43 in mice injected with LV-MUT-ATXN7 compared to non-injected mice or mice injected with LV-WT-ATXN7 (*n* = 3). **f** and **g** Optical densitometry was normalized according to the amount of actin loaded in the corresponding lane. A partition ratio was calculated and expressed as optical densitometry (arbitrary units) relative to the sample with highest value for the normalization control set at 1. Values are expressed as mean ± standard error (SEM) of the mean. *p ≤ 0.05 (one-way ANOVA). All data are from 3 mice/group. **h**–**k** Laser confocal microscopy showing co-aggregation of MUT ATXN7 (green) and FUS/TLS (red) in GCL in mice injected with LV-MUT-ATXN7 (69.6 ± 7.4 %), 12 weeks post-injection (I); in regions transduced with WT ATXN7 co-localization of WT ATXN7 with diffuse FUS/TLS in GCL, but no aggregation is observed (H). In the GCL transduced with MUT ATXN7, FUS/TLS is trapped into the ATXN7 inclusion and diffuse nuclear FUS/TLS fluorescence was decreased (see arrow) (K); in the contrary, in the non-transduced GCL (J) or in PCs transduced (H) with WT ATXN7 (green), FUS/TSL immunoreactivity was diffuse as well as WT ATXN7 in PCs (H). **l** Representative western-blot of cerebellar lysates shows reduced FUS/TLS levels (~50 %) (L) in mice injected with LV-MUT-ATXN7 compared to non-injected mice or mice injected with LV-WT-ATXN7 (*n* = 3). **m** Optical densitometry was normalized as indicated in panel F and G. Values are expressed as mean ± standard error (SEM) of the mean. *p ≤ 0.05 (Student’s *T* test). All data are from 3 mice/group. Bars: A–D: 20 μm; H and I: 10 μm; J and K: 3 μm

Next, we investigated whether in vivo ATXN7 accumulation in the nucleus affected two RNA binding proteins: the transactive response DNA binding protein 43-kDa (TDP-43) and the *Fused in sarcoma* (FUS/TLS). Immunofluorescence revealed that, in the LV-based model of SCA7, there was no clear co-aggregation between MUT ATXN7 and endogenous TDP-43, which remained diffuse in the nucleus (Fig. 6b). However, ~12 % of mutant ATXN7-positive aggregates contained pathological phosphorylated TDP-43 (p-TDP-43) (Fig. 6d), which was absent when truncated WT ATXN7 was overexpressed in the cell nucleus (Fig. 6c). On western-blots biopsies from regions infected with truncated MUT ATXN7, revealed by anti-phosphorylated TDP-43, a ~25-kDa band was increased ~1.6-fold compared to samples from non-injected mice or mice injected with LV-WT-ATXN7 (Fig. 6e and g). We next examined FUS/TLS expression in our LV-based model of SCA7. FUS/TLS and ATXN7 co-localized in ~70 % of intranuclear inclusions at 12 weeks post-infection (Fig. 6i). MUT ATXN7 overexpression also decreased the diffuse staining of endogenous FUS/TLS within the nucleus of granule cells (Fig. 6k); when truncated WT ATXN7 was overexpressed in PCs, FUS/TLS stained the cell nucleus, but did not form FUS/TLS-positive aggregates (Fig. 6h). Endogenous levels of FUS/TLS also decreased (~50 %) on western-blots of brain samples from cerebellar regions expressing truncated MUT ATXN7 (Fig. 6l and m) compared to samples from non-injected mice or WT ATXN7-injected mice.

Interestingly, similar results to those obtained in the LV-SCA7 model were found in *Atxn7*^*100Q/5Q*^ KI mice (Additional file 1: Figs. S4 and S5). Although more p-TDP-43-positive dots were detected in the nucleus of PCs of *Atxn7*^*100Q/5Q*^ KI mice compared to controls (Additional file 1: Fig. S4A), the levels of normal TDP-43 controls (data not shown) and phosphorylated TDP-43 (Additional file 1: Fig. S4B) in cerebellar lysates of *Atxn7*^*100Q/5Q*^ KI mice were similar. Interestingly, a ~25-kDa band labeled by the anti-phosphorylated TDP-43 antibody migrated more slowly in samples from *Atxn7*^*100Q/5Q*^ KI mice compared to control samples (Additional file 1: Fig. S4B). Moreover, these data were completed with additional immunofluorescence that revealed that FUS/TLS and mutant ATXN7 co-aggregated in the nucleus of PCs, accompanied by loss of endogenous FUS/TLS immunoreactivity compared to age-matched controls (Additional file 1: Fig. S5A). Western-blot analyses of whole cerebellar lysates from *Atxn7*^*100Q/5Q*^ KI mice also revealed a ~33 % decrease in the levels of soluble FUS/TLS protein (Additional file 1: Fig. S5B and C).

### MUT ATXN7 specifically traps the MBNL1 protein in the mouse cerebellum

**Fig. 7:**
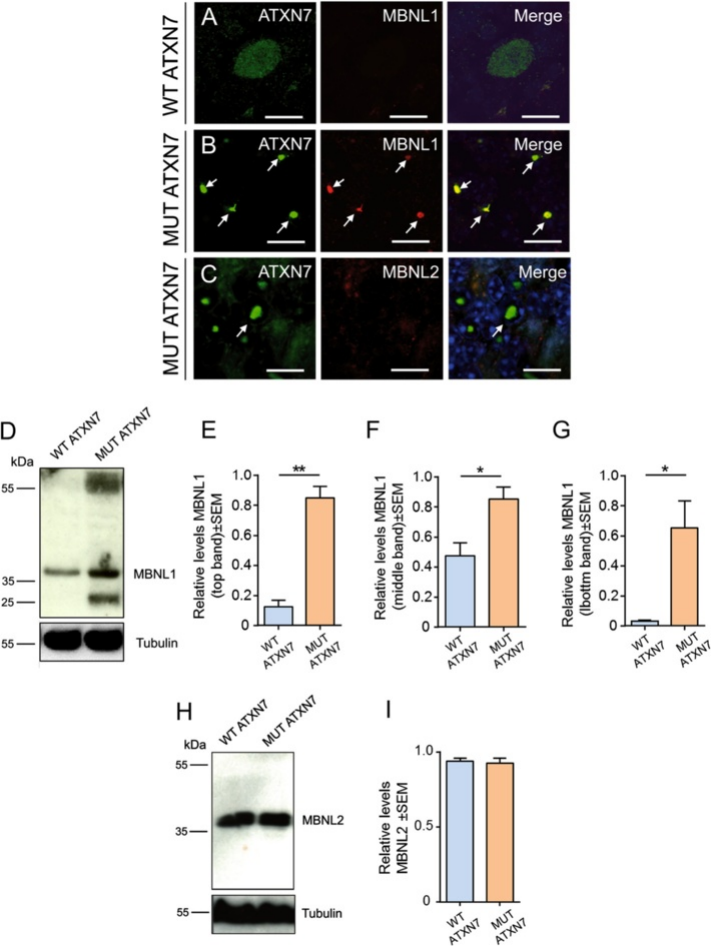
MUT ATXN7 specifically traps MBNL1 but not MBNL2 in the mouse cerebellum. **a**–**c** Laser confocal microscopy in cerebellum shows co-aggregation of MUT ATXN7 (green) and MBNL1 (red) in aggregates (~80 %) in the the GCL (B); no MBNL1 aggregation was observed in WT ATXN7-expressing PCs (A). The MBNL2 protein was not trapped in nuclear MUT-ATXN7 aggregates (C). **d** Representative western-blot of cerebellar lysates shows increased levels of the MBNL1 protein (55 kDa, 35 kDa and 25 kDa) in a mouse overexpressing MUT ATXN7 compared to a mouse overexpressing the control WT ATXN7 (*n* = 3). H) Western-blot: no differences in MBNL2 protein levels were observed in the cerebella of mice injected with LV-WT-ATXN7 or LV-MUT-ATXN7 (*n* = 3). Tubulin was used as a loading control. **e**, **f**, **g** and **i** Optical densitometry was normalized according to the amount of tubulin loaded in the corresponding lane. A partition ratio was calculated and expressed as optical densitometry (arbitrary units) relative to the sample with highest value for the normalization control set at 1. Values are expressed as mean ± standard error (SEM) of the mean. *p ≤ 0.05 (Student’s *T* test). All data are from 3 mice/group. Bars: A–C: 10 μm

To investigate whether the muscleblind-like proteins 1 and 2 (MBNL1 and/or MBNL2) RBPs could be implicated in SCA7 pathology we analyzed the effects of MUT ATXN7 overexpression on these specific RBPs in the cerebellum of LVs-injected mice. Immunofluorescence studies showed that MBNL1 co-aggregated within MUT ATXN7 inclusions in the PC nuclei of LV-MUT-ATXN7 injected mice (~80 %) (Fig. 7b), in contrast to the diffuse MBNL1 and ATXN7 staining in LV-WT-ATXN7 injected mice (Fig. 7a). Strikingly, no MBNL2 co-aggregation was found with ATXN7 in the cerebellum of LV-MUT-ATXN7 injected mice (Fig. 7c), strongly suggesting that MBNL1 is specifically trapped into MUT ATXN7 inclusions. We further analyzed the levels of expression of the MBNL1 and MBNL2 proteins by western-blot analysis of cerebellar lysates from mice injected with the LV-encoding truncated WT ATXN7 and MUT ATXN7. In mice injected with LV-MUT-ATXN7, we observed a slight increase in the levels of the MBNL1 ~ 35-kDa band relatively to LV-WT-ATXN7 injected mice (Fig. 7d). In addition, a strong increase in the levels of two additional bands around ˂25-kDa and ~55-kDa, maybe corresponding to different cerebellar MBNL1 isoforms [30], was observed in LV-MUT-ATXN7 injected mice compared to LV-WT-ATXN7 injected mice. (Fig. 7d–g). Interestingly, no difference was observed in the levels of the MBNL2 protein that showed a similar ~35-kDa doublet band in both the LV-WT-ATXN7 and LV-MUT-ATXN7 injected mice (Fig. 7h and i). Similar results were found by immunofluorescence in brain cerebellar slices (Additional file 1: Fig. S6A) and in western-blot analyses comparing MBNL1 and MBNL2 levels in cerebellar lysates from *Atxn7*^*100Q/5Q*^ KI mice and controls (Additional file 1: Fig. S6B-G), strongly supporting our findings and excluding a possible artefactual effect of LV-mediated ATXN7 overexpression .

### Immunoreactivity of TDP-43, FUS-TLS, MBNL1 and MBNL2 in the cerebellum of SCA7 patients

**Fig. 8:**
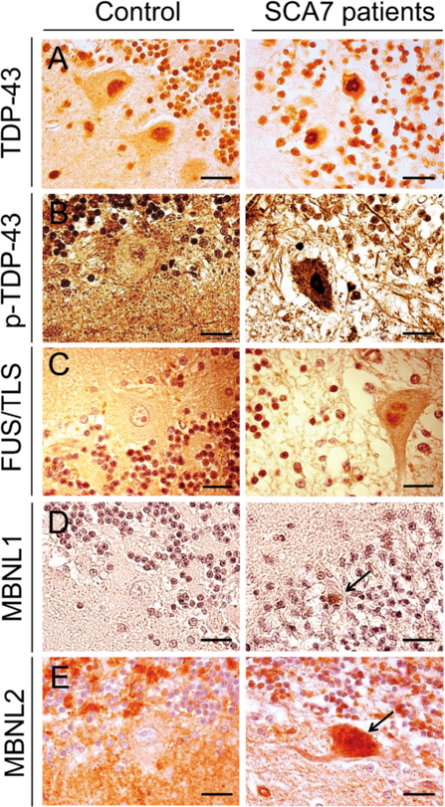
Immunoreactivity of TDP-43, FUS/TLS, MBNL1 and MBNL2 in the cerebellum of control and SCA7 patients. Representative immunohistochemically labelled cerebellar sections (counterstained with hematoxylin) from a SCA7 patient and a control with no neurological disease. **a** TDP-43 immunoreactivity was increased in the nucleus of PCs in the cerebellum of a SCA7 patient compared to control PCs where it remained diffuse with few TDP-43 dots. **b** Phosphorylated TDP-43 was strongly labeled in the nucleus of a PC of a SCA7 patient; only rare nuclear granules were observed in the nucleus of control PCs. **c** FUS/TLS shows increased immunoreactivity with FUS/TLS-positive small dots in the nucleus of a PC in a SCA7 patient compared to a control PC where FUS/TLS was almost not detectable in the nucleus. **d** Nuclear accumulation of the MBNL1 protein was higher in an atrophic PC in a SCA7 patient compared to PCs from a control. **e** MBNL2 diffuse immunoreactivity is increased in the nucleus and cytosol of a SCA7 PC, compared to control PCs where MBNL2 immunoreactivity is low in the cytoplasm and almost undetectable in the nucleus. Bars: 20 μm

To conclude the study, we investigated the immunoreactivity of TDP-43, FUS/TLS and muscleblind-like proteins 1 and 2 in the cerebellum of SCA7 patients and controls. In SCA7 patients, nuclear immunoreactivity for TDP-43 in PCs was more pronounced than in control PCs, where TDP-43 immunoreactivity was more diffuse (Fig. 8a). The phospho-TDP43 antibody strongly labeled the nucleus of PCs and granule cells in the cerebellum of SCA7 patients whereas labeling in PC nuclei and cytoplasm was only faint in controls (Fig. 8b). Immunolabeling of FUS/TLS was easily detectable in small granules in the nucleus of PCs in the cerebellum of SCA7 patients but not in controls (Fig. 8c). The MBNL1 protein accumulated in shrunken PC nuclei in SCA7 patients but not in controls, where it was almost not detectable (Fig. 8d). MBNL2 immunoreactivity increased in the nucleus, but also in the cytoplasm of SCA7 PCs compared to controls, where low MBNL2 immunoreactivity was cytosolic (Fig. 8e). These results confirm the abnormal accumulation of these RBPs in cerebellar PCs from SCA7 patients, which is in accordance with the LV-based model of SCA7.

## Discussion

When expressed in the cerebellum of transgenic/knock-in mice, mutant ATXN7 was reported to cause severe ataxic symptoms, but no significant cell loss [8, 15]. The local overexpression of mutant proteins using viral vectors has been successfully used to model neurodegenerative diseases [17, 18, 31–33]. In the present study, we demonstrated that LV-mediated delivery of mutant ATXN7 provided an alternative animal model of SCA7, with the development of an earlier, more robust and aggressive behavioral /histopathological phenotype than that observed in other SCA7 models [8, 14, 34]. It is, therefore, a time- and cost-effective animal model of SCA7 that partially avoids the limitations previously encountered, such as the slow development of the disease and the absence of neurodegeneration.

Remarkably, truncated fragments were shown to accumulate in SCA7 transgenic models, even when they express the full-length expanded form of ATXN7. An ∼ 55 kDa ATXN7 amino-terminal fragment was identified in both SCA7 transgenic mice and SCA7 patients [8]. Importantly, it has also been observed, in in vitro studies on SBMA, that full-length polyQ proteins aggregate, but at a much slower rate than their proteolytic fragments [35]. Protein proteolytic cleavage mediated by caspases produces small, polyQ-containing fragments with increased cellular toxicity [36]. The products of proteolytic cleavage are often found in aggregates, hallmarks of polyQ diseases, observed in both in vitro and in vivo models [37] and also in patients post-mortem tissue [38]. Therefore, we overexpressed, locally in the mouse cerebellum, an ATXN7 fragment similar in size to the cleaved fragments previously reported [8, 14, 34]. Indeed, the local LV-mediated overexpression of an ATXN7 fragment with an expanded polyQ repeat induced a cascade of events with progressive cell loss and a severe neuropathological phenotype. Furthermore, the local overexpression of mutant ATXN7 allowed us to dissect the specific contribution of SCA7-induced cerebellar pathophysiology, since other brain regions, such as the brainstem and *substantia nigra*, were also reported to be affected in this pathology [28]. This model may therefore be of use not only to dissect molecular mechanisms of SCA7 pathogenesis, but also to investigate in vivo new therapeutic strategies acting on cell degeneration and behavioral abnormalities. Importantly, SCA7 overexpression could be investigated at different time-points and SCA7 severity could be modulated by varying the dose of LV to be injected, as previously described in a LV-based rat model of SCA3 [17, 18].

Our first results showed that intracerebellar injection of LV encoding either wild-type or mutant truncated human ATXN7 resulted in strong and widespread transgene expression in the cerebellum. However, wild-type truncated ATXN7 was diffusely distributed throughout the Purkinje and granule cell nuclei, whereas mutant ATXN7 progressively accumulated in dense neuronal intranuclear aggregates and was depleted from the cytoplasm, as in previous SCA7 transgenic/knock-in models [8, 15]. Importantly, nuclear localization of mutated polyQ protein was proposed to be critical for the initiation of neuronal death in rodent SCA1 and SCA3 models [39, 40], suggesting that the nuclear environment of ATXN7 might be important for disease progression. Our study is in agreement with previous studies, as only transgenic nuclear mutant ATXN7 leads to PC loss, reduced thickness of the granule cell and molecular layers, 12 weeks post-injection, and co-aggregation with RBPs, whereas transgenic wild-type ATXN7, although nuclear, did not affect PC or other cerebellar layers.

In the present LV-based model of SCA7, the initial step in ATXN7 aggregation was visible 2 weeks post-injection in still well preserved PCs and GCL whereas, at the late stage of the disease, the mutant protein was completely aggregated in GCL but not in PCs, suggesting that: (i) the progressive accumulation of insoluble ATXN7 leads to time-dependent neuronal demise; (ii) the dynamics and toxicity of aggregate formation may vary considerably in different cell types from the same brain region; (iii) PCs appear to be more vulnerable than cells from the GCL, thus reproducing general features of human SCA7 pathophysiology [28]. Strong ATXN7 immunoreactivity is found widely throughout the GCL but not in PCs in SCA7 patients, whereas nuclear inclusions are infrequent, probably due to privileged PC loss [28]. Indeed, PCs can be injured by slight insults in comparison with other cells [41], and functional deficits of these cerebellar nerve cells, in particular, calbindin loss, are directly associated with compromised control of motor coordination [42, 43], such as in mice infected with mutant ATXN7. In this LV-SCA7 model we could also highlight the loss of synaptic markers, which has not been previously demonstrated in animal models of SCA7, as well as the increased immunoreactivity found in astrocytic and glial cells that may be interpreted as associated to pathogenesis and/or an adaptative immune response against excitotoxicity induced by mutant ATXN7.

This LV-based model of SCA7 also enabled investigation of neurodegenerative mechanisms. Indeed, the most studied mechanisms of pathogenesis were centered on the abnormal aptitude of mutant proteins to attract cellular proteins, such as ubiquitin, proteasome components, transcription factors and chaperones, in aggregates causing loss of the homeostasis by means of primary proteinopathy. As in our LV-based model of SCA7, several SCA7 rodent models [14, 15], which replicate many features of the human condition, and brains from SCA7 patients [11, 44] display abundant inclusions that consistently stain positively for proteasome subunits, ubiquitin, and molecular chaperones. Indeed, overexpressed mutant ATXN7 sequesters autophagy-related proteins, as previously described in a SCA7 knock-in model [29], and molecular chaperones (data not shown), important for the maintenance of cell homeostasis, depleting them from neurons. This clearly reflects impaired protein clearance pathways.

In the last few years, it has been shown that RNA-related mechanisms may play an important role in polyQ disorders [13]. However, information is scarce concerning events implicating specific RBPs in animal models of polyQ disorders, including SCA7. Our findings suggest a direct link between impaired protein degradation and accumulation of misfolded ATXN7 that sequesters other molecules, such as particular RBPs, depleting them [21] and/or promoting the expression of aberrant, misregulated isoforms [45]. Studies in in vitro models and transgenic mouse models demonstrate that expanded polyQ proteins are more toxic when translocated into the nucleus [46], suggesting that the nucleus is a crucial site of pathogenesis in polyQ disorders.

Recently, the RBPs FUS/TLS and TDP-43 were shown to co-localize in nuclear Gems implicated in spliceosome maintenance [47]. FUS/TLS shares several structural and functional properties with TDP-43; both are genetically related to ALS and FTLD, and are nuclear proteins with RNA and DNA binding abilities that play a role in RNA splicing- reviewed in [48]. FUS/TLS binds strongly to SCA1, SCA2, HD and DRPLA aggregates [20, 21, 49]. In addition, several variants of the gene have been identified as risk factors for ALS and rare forms of FTLD [50], suggesting that FUS/TLS plays a role in neurodegenerative diseases. In our LV-based model and in SCA7^100Q/5Q^ knock-in mice, we observed a strong co-localization between ATXN7 and FUS/TLS in aggregates. To our knowledge, this is the first study reporting an association between ATXN7 and FUS/TLS and a decrease in FUS/TLS expression that may result from sequestration in inclusions. This suggests that an inadequate supply of this protein could result in abnormal transcription, RNA processing and transport, and potentially cause instability of dendritic spines, as observed in R6/2 HD mice [51]. Notwithstanding, further studies are needed to elucidate the exact mechanism. Importantly, primary neurons from FUS/TLS-deficient mice have a decreased number of spines, and those remaining have a non-standard morphology [52], indicating that FUS/TLS is important for neuronal function. Remarkably, we also show that FUS/TLS is preferentially trapped in ATXN7 inclusions compared to TDP-43. In accordance with our results, FUS/TLS co-localized with polyQ proteins in neuronal intranuclear inclusions in SCA2 whereas TDP-43 did not [53]. TDP-43 also co-localized with huntingtin in dystrophic neurites and intracellular inclusions, but not in intranuclear inclusions [54]. Secondary TDP-43 proteinopathies have been described in other CAG repeat disorders, such as SCA2 [55], SCA3 [56] and HD [54], suggesting that these disorders might share with ALS some pathological mechanisms involving TDP-43.

In our LV model and in SCA7 patients, a few neurons stained positively for p-TDP43 pS409/410, which was confirmed to be a valuable tool for detecting abnormal TDP-43 in patients and to evaluate TDP-43 proteinopathies in animal models of neurodegenerative disorders [57]. Furthermore, phosphorylation of aggregated TDP-43 at S409/410 is a defining hallmark of TDP-43 proteinopathies, including ALS and FTLD-TDP [58, 59]. Phosphorylation of TDP-43 at serines 409 and 410 was recently reported to promote TDP-43 toxicity in vivo [60]. Excitingly, in SCA7^100Q/5Q^ knock-in mice, we observed a hyperphosphorylated ~25-kDa species that could potentially be generated from alternative translational or splicing mechanisms, as previously suggested [57, 61]. Further studies will be needed to better understand the contribution of TDP-43 to SCA7 pathogenesis.

Nevertheless, whether the accumulation of the RBP MBNL1 contributes to pathology or is simply an epiphenomenon, in addition to other features of polyQ pathophysiology, will need further investigations. In this LV model of SCA7, as well as in *Atxn7*^*100Q/5Q*^ KI mice and SCA7 patients, MBNL1 co-localized with ATXN7 inclusions. In addition, the specific accumulation of MBNL1 in ATXN7 inclusions was associated with an increased level of MBNL1 in both SCA7 mouse models compared to wild-type mice. In contrast, MBNL2 did not co-localize with ATXN7 in inclusions and its level remains unchanged. The consequences of MBNL1 accumulation in abnormal proteinaceous inclusions remain to be elucidated. It may be associated with neurotoxicity, although recent data also suggest that MBNL proteins might be potential modifiers of polyQ disorders, given that MBNL1 suppresses the expression of polyQ-containing proteins [62]. The mechanism leading to the specific accumulation of MBNL1 in ATXN7 inclusions remains unknown. It would be of interest to modulate the LV-SCA7 model by overexpressing or knocking-out these RBPs to assess their impact on SCA7 pathology, in particular on aggregate formation, but also to determine how they affect neuronal markers and inflammation. To conclude, the complete elucidation of these mechanisms will be important for understanding SCA7 and related polyQ disorders and the development of potential therapeutics.

## Conclusions

This study validates a novel LV-based SCA7 mouse model, in which strong and sustained expression of MUT ATXN7 in the cerebellum was found sufficient to generate motor defects. This model can be further exploited to better understand the importance of RBPs in RNA/protein-mediated neuropathology and to evaluate potential therapies [63, 64] for SCA7 and other polyQ disorders.

## Methods

### Lentiviral vector production

The HIV-1-derived pRRL-SIN-cPPT-PGK-ATXN7T-10Q-GFP-WPRE and pRRL-SIN-cPPT-PGK-ATXN7T-100Q-GFP-WPRE transfer plasmids were constructed by sub-cloning two previously described truncated versions of the human SCA7 (Gene ID: 6314) cDNA (amino acids 1–232) [11], encompassing 10 glutamines (wild-type, WT ATXN7-10Q) or 100 glutamines (mutant, MUT ATXN7-100Q) into a HIV-1 derived vector plasmid, containing a GFP reporter gene at the 5’ end (pRRL-SIN-cPPT-PGK-GFP-WPRE) and an added NLS derived from the SV40, placed under control of the phosphoglycerate kinase 1 (PGK-1) promoter.

Stocks of VSV-G pseudotyped self-inactivating lentiviral vectors were produced in 293 T cells using a four-plasmid system as described previously [65]. The LV particles were concentrated by ultracentrifugation and resuspended in phosphate-buffered saline (PBS). Vector titers (expressed in TU/ml) were determined by quantitative PCR amplification (SybRGreen detection) 4 days post-transduction in HCT116 human fibroblastic cells with serial dilutions of vector. Viral stocks were stored at−80 °C until use.

### Animals

Four-week-old female C57BL/6 mice (Charles River, Les Oncins, France) weighing ~15–17 g were injected into the cerebellum with LV encoding truncated wild-type or truncated mutant human ataxin-7. Heterozygous SCA7^100Q/5Q^ knock-in female mice (hereafter called SCA7 KI mice) carrying 100 CAG repeats in the mouse *Sca7* locus [16] on the pathological allele derived from the SCA7^266Q/5Q^ [15] were also used. SCA7 KI mice were analyzed at 12 months of age (*n* = 4) (late stage disease) and compared to 12-month-old (*n* = 4) wild-type littermates. Animals were housed in a temperature-controlled room and maintained on a 12 h light/dark cycle. Food and water were available *ad libitum*. The experiments were carried out in accordance with the European Community Council directive (86/609/EEC) for the care and use of laboratory animals and were approved by the Commission Génie Génétique of the French Ministry for Scientific Research and Education.

### In vivo injection of lentiviral vectors in the mouse cerebellum

Wild-type mice were anesthetized by administration of ketamine (75 mg/kg, i.p.) and xylazine (10 mg/kg, i.p.) and injected stereotactically into the vermis with LVs encoding the green fluorescent protein (GFP) (*n* = 3), human truncated wild-type ataxin-7 (WT ATXN7; ATXN7-10Q) (*n* = 9) or human truncated mutant ataxin-7 (MUT ATXN7; ATXN7-100Q) (*n* = 11). LVs were injected into the mouse cerebellum through a 34-gauge blunt-tip needle linked to a Hamilton syringe (Hamilton, Reno, NV, USA) by a polyethylene catheter. The viral suspensions (3×10^6^ TU/ml) were injected at 0,25 μl/min using an automatic injector (Stoelting Co., Wood Dale, USA). Three μl of LV suspension were injected at the following coordinates:−6.5 mm *rostral to bregma*, 0 mm *lateral to midline*, and−1.1 mm *ventral from the dura mater*, with the mouth bar set at 0. After injection, the syringe needle was left in place for an additional 5 min before being slowly raised. The skin was closed using a 6-0 Prolene® suture (Ethicon, Johnson and Johnson, Brussels, Belgium).

### Histology

Two weeks (*n* = 3 for LV-GFP; *n* = 3 for PBS; *n* = 3 for LV-WT ATXN7; *n* = 3 for MUT ATXN7) or 3 months (*n* = 6 for LV-WT ATXN7; *n* = 8 for MUT ATXN7) after LV injection into the cerebellum, the animals were given an overdose of sodium pentobarbital and perfused transcardially with a solution of 0.1 M phosphate-buffered saline (PBS) and then with 4 % paraformaldehyde (PFA) in PBS. The brains were removed, post-fixed in 4 % PFA in PBS and processed as described previously [27]. See also Additional file 2.

### Immunohistochemistry

Immunofluorescence and 3,3’-Diaminobenzidine (DAB) staining were carried out as previously described [27]. See also Supplementary materials and methods.

### Human SCA7 brain samples

Post-mortem cerebellar tissue from two SCA7 patients with morphologically and genetically confirmed SCA7 (10 and 36 years of age at death, with 85 and 49 CAG repeats on the mutant allele in peripheral blood, respectively) and two controls with no evidence of neurological disease (57 and 72 years of age at death) were obtained from the Department of Neuropathology of the Pitié-Salpêtrière Hospital (Paris).

### Primary antibodies

See Table 1.

**Table 1:** Antibodies used in western-blot (WB) and immunohistochemical (IHC) analyses

Primary antibodies	Source/Reference catalog	WB	IHC
rabbit anti-ataxin-7	Thermo Scientific/(PA1-749)	1:2000	1:5000
rabbit-anti-ubiquitin	Dako/(Z0458)	–	1:2000
mouse anti-ataxin-7 (1C1)	Provided by Dr Didier Devys	1:5000	1:5000
mouse anti-Calbindin D-28 K	Swant/(300)	–	1:4000
mouse anti-microtubule-associated Protein 2 (MAP2)	Sigma-Aldrich/(M1406)	–	1:2000
mouse anti-synaptophysin	Abcam/(ab32594)	–	1:250
mouse anti-SNAP-25	Abcam/(ab24737-250)	–	1:1000
rabbit-anti-PSD-95	Abcam/(ab18258)	–	1:1000
rabbit anti-Glial Fibrillary Acidic Protein (GFAP)	Dako/(Z0334)	–	1:2000
rabbit anti-ionized calcium binding adapter molecule 1 (Iba1)	Wako/(019-19741)	–	1:3000
rat anti-CD11b/Mac-1	AbD Serotec/(MCA74G)	–	1:1000
rabbit anti-GFP	Abcam/(ab6556)	–	1:5000
rabbit cleaved caspase-3 (Asp175)	Cell Signaling/(9664S)	–	1:500
mouse anti-neurofilament 70 KDa	Millipore/(MAB1615)	–	1:1000
rabbit anti-phospho-TDP-43 (pS409/410)	Cosmo Bio/(CAC-TIP-PTD-P01)	1:1000	1:3000
rabbit anti-TARDBP (TAR DNA-binding protein 43)	Proteintech/(10782-2-AP)	–	1:3000
Mouse anti-FUS/TLS(4H11) (Fused in Sarcoma/Translocated in Sarcoma)	Santa Cruz/(sc-47711)	1:1000	1:500
rabbit-anti-MBNL1 (Muscleblind-like protein 1)	Santa Cruz/(sc-47740)	1:1000	1:500
rabbit-anti-MBNL2 (Muscleblind-like protein 2)	Abcam (ab171551)	1:2000	1:500
rabbit-anti-MBNL2	Abcam (ab108519)	–	1:500
rabbit anti-tubulin	Abcam/(ab134185)	1:5000	–
rabbit anti-iNOS (Nitric oxide synthase)	Cayman Chemicals/(160862)	–	1:400
rat anti-CD68	Abd serotec/(MCA1957)	–	1:500
rabbit anti-TGF-ß (Transforming growth factor beta)	Abcam/(ab66043)	–	1:1000
rabbit anti-TREM2 (Triggering receptor expressed on myeloid cells 2)	R&D systems/(MAB1729)	–	1:500
goat anti-vimentin	Santa Cruz/(sc-7557)	–	1:500
rabbit anti-p62/SQSTM1 (sequestosome 1)	BD Biosciences/(610832)	–	1:500
mouse anti-actin	Abcam/(ab9484)	1:4000	–
mouse anti-SMI-25	Covance/*(SMI-25R)*	–	1:2000

### Behavioral testing

#### Rotarod

Motor coordination and balance were estimated using a five station mouse rotarod (Bioseb, Vitrolles, France). Mice were first trained at increasing speed up to a constant speed of 16 rpm. Subsequently, the latency to fall was recorded automatically by incremental speed starting at 4 rpm and accelerating over a 2 min period up to 40 rpm, using break technology. Each mouse was tested 3 times per day (with at least 15 min rest between the trials) three times a week, at 2, 4, 8 and 12 weeks after LV injection. Statistical analysis of obtained data was performed by calculating the mean values of each trial for each group (mean of 3 daily trials per week with three trials per day). Data are presented as the mean ± SD of the latency to fall in each group and differences between groups were analyzed at each time using one-way ANOVA followed by a *post-hoc* Fisher’s test.

#### Locotronic test

The locotronic apparatus was used to test fine motor coordination when walking. The mice crossed a 75 × 5 × 20 cm flat ladder with bars (7 mm in diameter), which were set 2 cm apart. Infrared photocell sensors situated above and below the bars monitored paw errors. The locotronic apparatus was supported by software that automatically sorted paw errors and directly calculated the time of the course. The rate of missteps and the time of the course were assessed in three trials/day for three consecutive days, with 20 min rest between trials. The tests were performed at 2, 4, 8 and 12 weeks post-injection. Statistical analysis of obtained data was performed by calculating the mean of three trials per day over 3 days for each group. Data are presented as the mean ± SD. Differences between groups were analyzed at each time using one-way ANOVA followed by a *post-hoc* Fisher’s test.

#### Spontaneous activity

An actimeter system (Activmeter; Bioseb, Vitrolles, France) was used to measure vibrations within the cage to assess locomotion. The distance covered, movements and average speed were measured over a period of 45-min period. The tests were performed at 12 weeks post-injection. Data are presented as the mean ± SD. Differences between groups were analyzed at each time using one-way ANOVA followed by a *post-hoc* Fisher’s test.

#### Western-blot

Mouse cerebella/brainstem were collected and frozen at−80 °C. Protein extracts (100 μg) were resolved on 7.5 % (ataxin-7), 12 % (FUS/TLS, MBNL1, and MBNL2) or 15 % [phospho-TDP-43 (pS409/410)] SDS-polyacrylamide gels. Western-blotting procedures were performed as previously described [27]. Films were scanned and optical densities (OD) were measured using Quantity One 1D image analysis software (version 4.4; Biorad, Hercules, CA, USA). The optical densities were normalized with respect to a “standard protein” (actin or tubulin) migrating in the same lane. A partition ratio was calculated and normalized with respect to the sample with the highest value defined as 1.

### Statistical analysis

Statistical analyses for evaluation of differences between groups for calbindin immunostaining, cresyl violet staining, and spontaneous motor activity in the actimeter were performed using One-way ANOVA followed by a *post-hoc* Fisher’s test. Statistical analysis for behavior assessment in the rotarod and locotronic tests was performed using Two-way ANOVA followed by a post-hoc Fisher’s test. Optical densities (OD) of scanned western-blot films and percentage of marker co-localization in IF studies were analyzed using the Student’s *t*-test or One-way ANOVA followed by a post-hoc Fisher’s test. Significance thresholds were set at *p* < 0.05 for all tests.

## Abbreviations

ALS, amyotrophic lateral sclerosis; ATXN7, ataxin-7; DNA binding protein 43 kDa; FUS/TLS, Fused in sarcoma; HD, Huntington’s disease; KI, knock-in; LV, lentiviral vector; MBNL1, muscleblind-like 1; MBNL2, muscleblind-like 2; NIIs, neuronal intranuclear inclusions; PC, Purkinje cell; polyQ, polyglutamine; RBPs, RNA-binding proteins; SCA7, spinocerebellar ataxia type 7; TDP-43, transactive response

## Additional files

Additional file 1: Fig. S1. Lentiviral-mediated overexpression of the Green Fluorescent Protein (GFP) in the mouse cerebellum. **Fig. S2.** Overexpression of truncated MUT ATXN7 in the mouse cerebellum induces the formation of ubiquitinated ATXN7 aggregates **Fig. S3.** Lentiviral-mediated overexpression MUT ATXN7 in the mouse cerebellum, at 2 weeks post-infection (early time point). **Fig. S4.** Phosphorylated TDP-43 expression in the cerebellum of A*txn7* ^*100Q/5Q*^ KI mice. **Fig. S5.** FUS/TLS is trapped in ATXN7 aggregates in A*txn7* ^*100Q/5Q*^ KI mice. **Fig. S6.** MBNL1 and MBNL2 immunoreactivity and expression in the cerebellum of *Atxn7* ^*100Q/5Q*^ KI mice. (PDF 1053 kb)

Additional file 2: Supplementary Materials and Methods- Alves et al. (DOCX 20 kb)
