# Supplementary material for: Lentiviral vector-mediated overexpression of mutant ataxin-7 recapitulates SCA7 pathology and promotes accumulation of the FUS/TLS and MBNL1 RNA-binding proteins
Source: Mol Neurodegener. 2016 Jul 28;11:58. doi: 10.1186/s13024-016-0123-2 (PMC4964261; doi:10.1186/s13024-016-0123-2)
Supplement: Supplementary file 1 — Lentiviral-mediated overexpression of the Green Fluorescent Protein (GFP) in the mouse cerebellum. Fig. S2. Overexpression of truncated MUT ATXN7 in the mouse cerebellum induces the formation of ubiquitinated ATXN7 aggregates Fig. S3. Lentiviral-mediated overexpression MUT ATXN7 in the mouse cerebellum, at 2 weeks post-infection (early time point). Fig. S4. Phosphorylated TDP-43 expression in the cerebellum of Atxn7 100Q/5Q KI mice. Fig. S5. FUS/TLS is trapped in ATXN7 aggregates in Atxn7 100Q/5Q KI mice. Fig. S6. MBNL1 and MBNL2 immunoreactivity and expression in the cerebellum of Atxn7 100Q/5Q KI mice. (PDF 1053 kb) [file 13024_2016_123_MOESM1_ESM.pdf]

Supplementary Figure 1

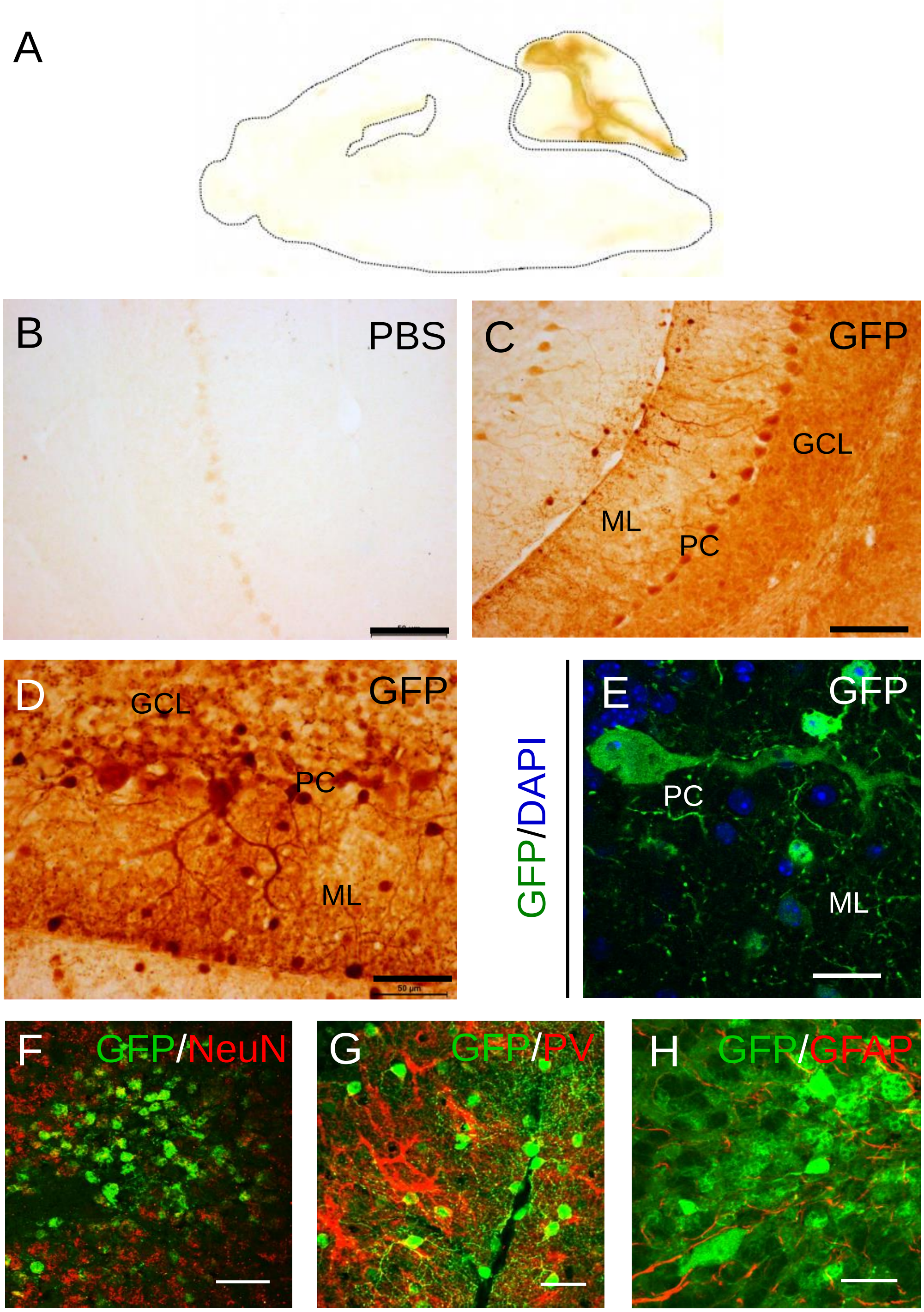

Supplementary Figure 1- Lentiviral-mediated overexpression of the Green Fluorescent Protein (GFP) in the mouse cerebellum.

A) Representative sagittal mouse brain slice showing GFP immunoreactivity in the cerebellum, locally injected with LV encoding GFP. GFP immunoreactivity in different cell layers (Purkinje cell layer (PC), granular cell layer (GCL) and molecular layer (ML) of the mouse cerebellum (C and D), 3 months after lentiviral mediated delivery in the cerebellar vermis. In control mice injected with PBS, no GFP immunoreactivity is detected (B). Laser confocal microscopy showing GFP immunoreactivity in a PC and in the ML. Nuclei were counterstained with 4',6-diamidino-2-phenylindole (DAPI). F) Laser confocal microscopy showing co-localization between the GFP protein (green) and neurons (NeuN staining; red) in the GCL; G) double staining for GFP (green) and parvalbumin (PV; red) shows transduction of stellate cells and respective arborisations (green) of the ML; H) No evident co-localization is observed between GFP (green) and the astrocytic marker GFAP (red) in the GCL. Representative data from 3 mice/group. Bars: B and C: 50 µm, D: 20 µm and E: 20 µm; F: 30 µm; G: 30 µm; G: 30 µm.

Supplementary Figure 2

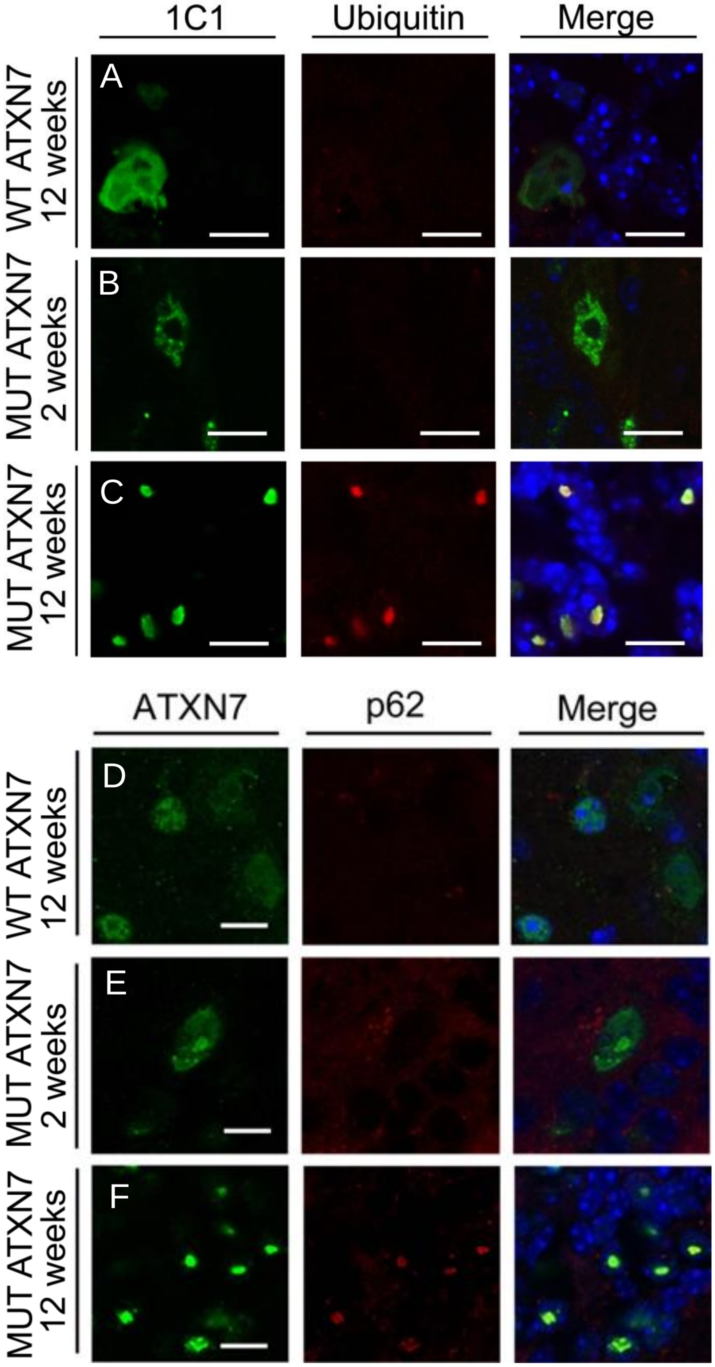

Supplementary Figure 2- Overexpression of truncated MUT ATXN7 in the mouse cerebellum induces the formation of ubiquitinated ATXN7 aggregates.
Large ubiquitin-positive inclusions (~93%) and p62-positive inclusions (~80%) co-localize with ATXN7 aggregates in cerebellar granular cells in animals injected with LV encoding truncated mutant human ATXN7, 12 weeks post-injection (C and F, respectively). At 2 weeks, truncated MUT ATXN7 has immunoreactivity in Purkinje cells and granule cells with the presence of small aggregates (B and E, respectively); no ubiquitin and p62 accumulation is observed in mice injected with LV-WT-ATXN7, at 12 weeks post-injection (A and D, respectively). Nuclei were counterstained with 4',6-diamidino-2-phenylindole (DAPI). Representative data from 3-5 mice/group. Bars: 10 µm.

Supplementary Figure 3

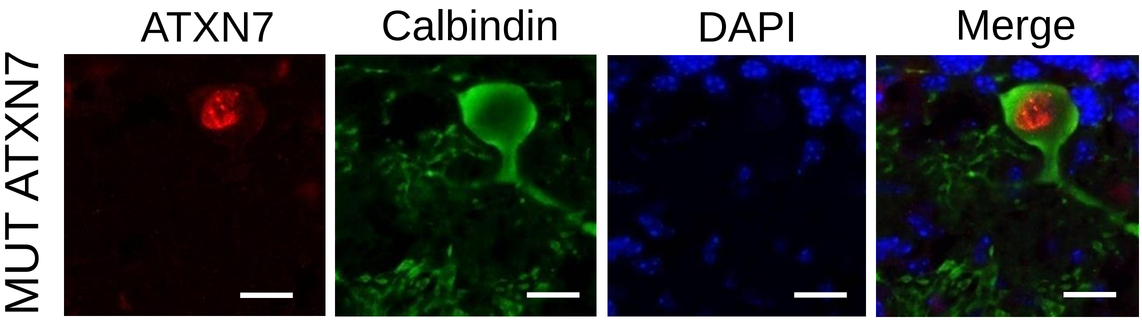

Supplementary Figure 3- Lentiviral-mediated overexpression MUT ATXN7 in the mouse cerebellum, at 2 weeks post-infection (early time point).
MUT ATXN7 (red) has diffuse immunoreactivity in the nucleus of Purkinje cells stained with calbindin (green), where small early aggregates (in red) are present Nuclei were counterstained with 4',6-diamidino-2-phenylindole (DAPI). Representative data from 3 mice/group. Bar: 10 µm.

Supplementary Figure 4

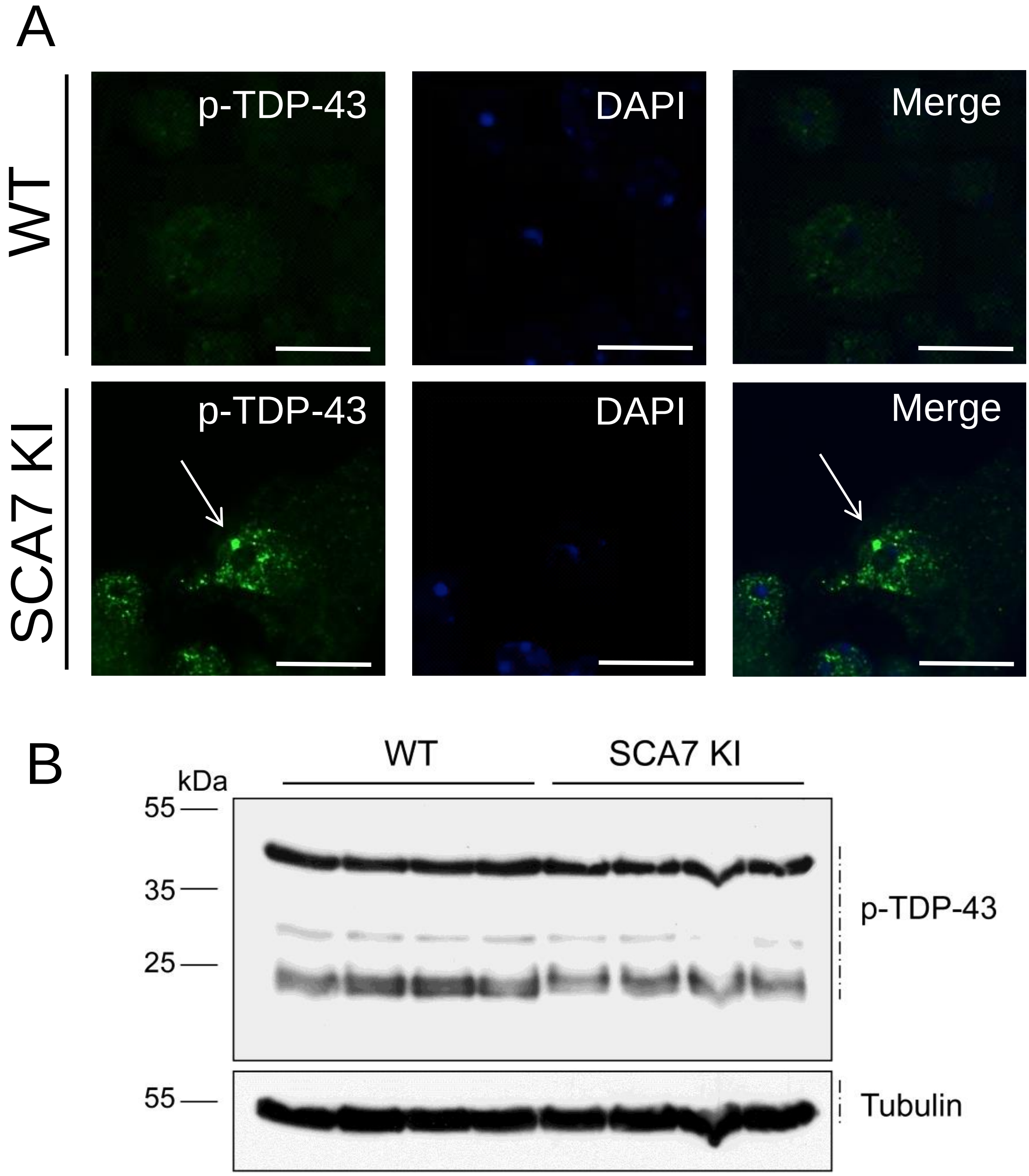

Supplementary Figure 4- Phosphorylated TDP-43 expression in the cerebellum of *Atxn7$^{100Q/5Q}$* KI mice.

A) Laser confocal microscopy showing phosphorylated TDP-43-positive dots in Purkinje cells from *Atxn7$^{100Q/5Q}$* KI mice, relative to age-matched littermates. B) Representative western-blot of whole cerebellar lysates from *Atxn7$^{100Q/5Q}$* KI mice and control littermates probed with the anti-phosphorylated TDP-43 antibody; no differences were found in the levels of normal TDP-43 (~43-kDa) in cerebellar lysates from *Atxn7$^{100Q/5Q}$* KI and wild-type mice; however a ~25-kDa band shows reduction in gel mobility in biopsies from *Atxn7$^{100Q/5Q}$* KI mice. All data are from 12-month-old mice (4 mice/group). Bars: 10 µm.

Supplementary Figure 5

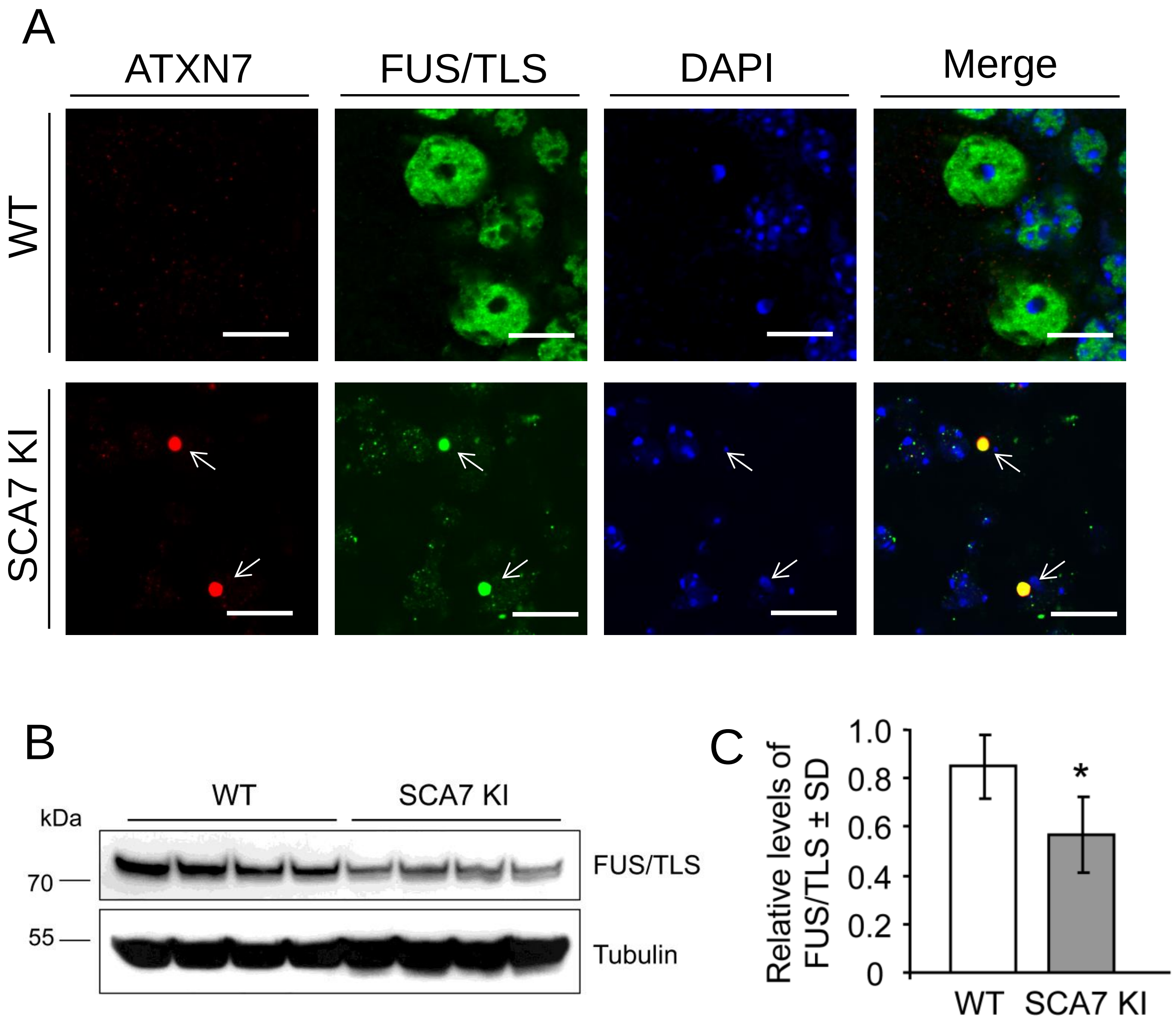

Supplementary Figure 5- FUS/TLS is trapped in ATXN7 aggregates in *Atxn7^100Q/5Q^* KI mice. A) Laser confocal microscopy showing diffuse immunoreactivity of FUS/TLS in the nucleus of Purkinje cells in control age-matched littermates. In *Atxn7*$^{100Q/5Q}$ KI mice, FUS/TLS is trapped in ATXN7 aggregates in the nucleus of Purkinje cells (see arrows). B) Representative western-blot of whole cerebellar lysates of *Atxn7*$^{100Q/5Q}$ KI mice showing decrease in the soluble levels of rodent FUS/TLS relative to wild-type controls. Tubulin was used as a loading control. C) Optical densitometry was normalized according to the amount of tubulin loaded in the corresponding lane. A partition ratio was calculated and expressed as optical densitometry (arbitrary units) relative to the sample with highest value for the normalization control set at 1. Values are expressed as mean ± standard deviation (SD) of the mean. *p≤0.05 (Student's T test). All data are from 12-month-old mice (4 mice/group). Bars: 10 µm.

Supplementary Figure 6

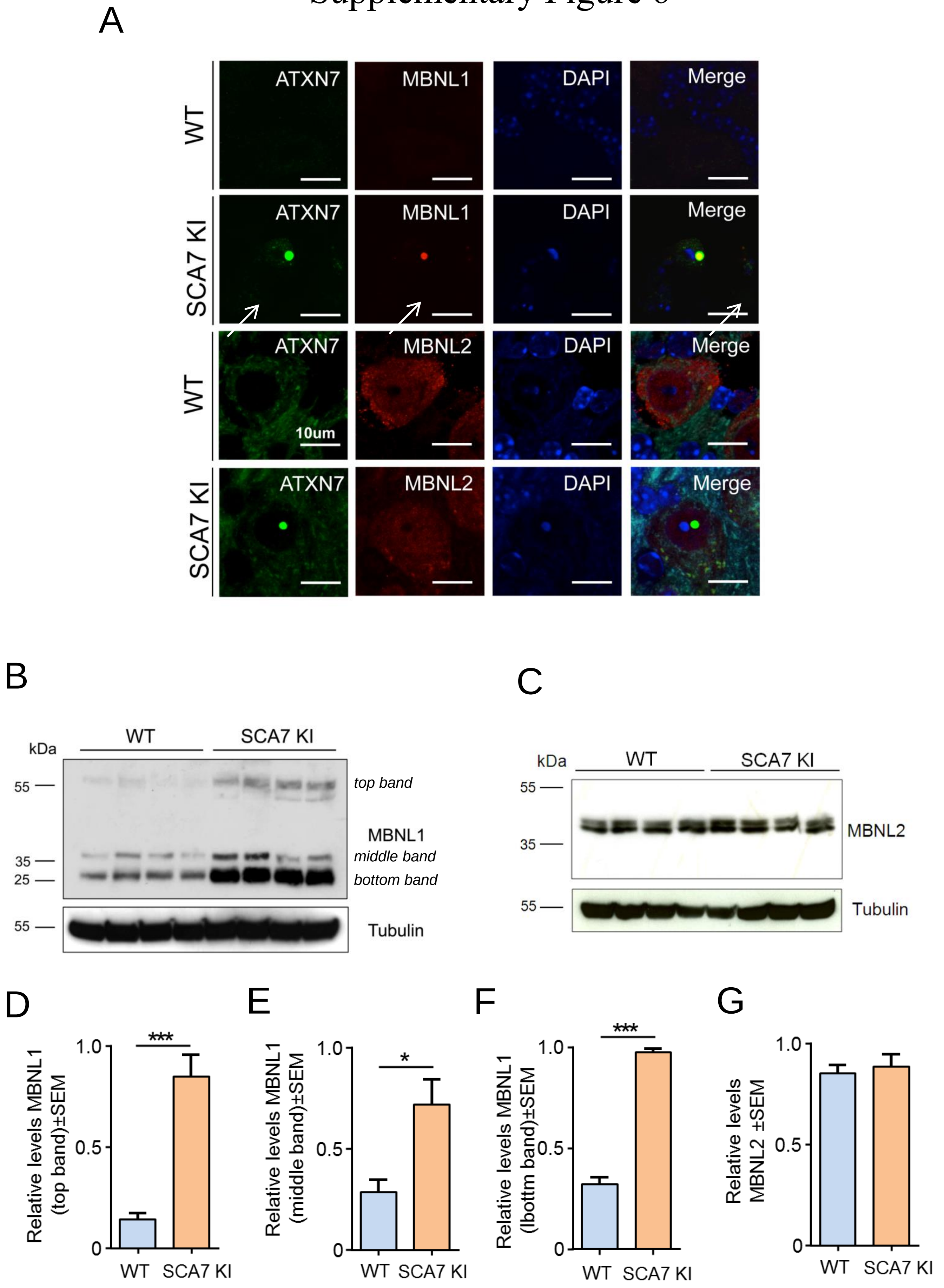

Supplementary Figure 6- MBNL1 and MBNL2 immunoreactivity and expression in the cerebellum of *Atxn7*$^{100Q/5Q}$ KI mice.

A) Laser confocal microscopy showing robust co-localization between MBNL1 and ATXN7 in nuclear aggregates of cerebellar Purkinje cells in *Atxn7*$^{100Q/5Q}$ KI mice (see arrows); no accumulation of MBNL1 is observed in age-matched littermates. No MBNL2 aggregation is observed in the Purkinje cells of *Atxn7*$^{100Q/5Q}$ KI mice, despite the presence of ATXN7 aggregates (arrow); in both *Atxn7*$^{100Q/5Q}$ KI mice and control littermates MBNL2 has diffuse immunoreactivity. B) Representative western-blot of whole cerebellar lysates of *Atxn7*$^{100Q/5Q}$ KI mice, probed with the anti-MBNL1 antibody showing increased expression of MBNL1-positive bands (~25-kDa band, ~35-kDa band and ~55-kDa band; top, middle and bottom bands, respectively) compared to age-matched littermates. C) Representative western-blot of whole cerebellar lysates of *Atxn7*$^{100Q/5Q}$ KI mice and age-matched littermates showing no differences in the levels of the MBNL2 protein. Tubulin was used as a loading control. All data are from 12-month-old mice (4 mice/group). D-G) Optical densitometry was normalized according to the amount of tubulin loaded in the corresponding lane. A partition ratio was calculated and expressed as optical densitometry (arbitrary units) relative to the sample with highest value for the normalization control set at 1. Values are expressed as mean ± standard error of the mean (SEM) of the mean. *$p \leq 0.05$; *** $p \leq 0.001$ (Student's T test). Bars: 10 µm.
