# Supplementary material for: Lentiviral vector-mediated overexpression of mutant ataxin-7 recapitulates SCA7 pathology and promotes accumulation of the FUS/TLS and MBNL1 RNA-binding proteins
Source: Mol Neurodegener. 2016 Jul 28;11:58. doi: 10.1186/s13024-016-0123-2 (PMC4964261; doi:10.1186/s13024-016-0123-2)
Supplement: Supplementary file 2 — Supplementary Materials and Methods- Alves et al. (DOCX 20 kb) [file 13024_2016_123_MOESM2_ESM.docx]

**Supplementary Materials and Methods- Alves et al.**

**Histology**

After an overdose of sodium pentobarbital, the mice were perfused transcardially with 4% paraformaldehyde (PFA) in 0.1M phosphate-buffered saline (PBS). The brains were removed and post-fixed in 4% PFA in PBS for 24 h and cryoprotected by incubation in 15% sucrose/0.1 M phosphate buffer for 48 h then frozen. Sagittal sections (20 µm) of the entire brain were cut at -20°C on a sliding cryostat (Cryocut 1800, Leica Microsystems AG, Glattbrugg, Switzerland), collected in anatomical series on microscope slides and stored at -80°C. Alternatively, the brains were dehydrated in increasing concentrations of ethanol, embedded in paraffin (Shandon Excelsior tissue processor, Thermo Scientific) and cut in 5 µm sections.

**Cresyl violet staining**

Premounted sections were immersed for 1 min in 0.1% cresyl violet, differentiated in acetate buffer pH 3.8-4 (2.72% sodium acetate and 1.2% acetic acid; 1:4 v/v), dehydrated in graded ethanol and xylene solutions, and coverslipped with Eukitt® (O. Kindler GmbH & CO. Freiburg, Germany).

**Flurojade B staining**

Sections were stained with **FluoroJade-B** (Millipore, Temecula, CA, USA), an anionic fluorescein derivative which stains neurons undergoing degeneration. The sections were first washed in water and then mounted on silanized glass slides, dehydrated, and stained according to the supplier's manual.

**NeuroTrace staining**

Sections were stained with NeuroTrace® Fluorescent Nissl Stain (**Life Technologies,** Saint Aubin, France), a marker of the physiological state of the neuron, according to the supplier's manual.

**Counting of Purkinje cells and analysis of the cerebellar granule and molecular layers**

Bright field images of calbindin- or cresyl violet-stained section were taken with an Olympus CKX41 light microscope, at 10X magnification bright-field microscopy, and analyzed with ImageJ software (NIH, USA). The total number of calbindin-positive cells in the cerebellum was determined blindly by analyzing eight to ten regularly spaced sagittal sections per animal (200 µm between 20 µm thickness sections), to sample the whole extent of the injected region. The thickness of the molecular and granule layers was determined blindly in the same region on 8 cresyl violet-tained sections. Data are presented as the mean ± SD.

**Image acquisition**

Images of immunostained sections were acquired with LAS V3.8 (Leica) software, at room temperature, with a brightfield Leica DM 4000B microscope equipped with 40×/0.75 and 100×/1.30 lenses and a Leica DFC500 digital camera. Confocal images were acquired with an Olympus BX 61 microscope equipped with 60x/1.35 lens and Fluoview FV-1000 image acquisition system. Photographs for comparative analyses were taken under identical conditions of acquisition, and adjustments of brightness and contrast were applied uniformly to all images.

**Immunohistochemistry**

Endogenous peroxidase was quenched for 20 min at RT in a PBS/0.1% Triton X-100 solution containing 10% methanol and 0.003% H_2_O_2_. Brain sections were then incubated sequentially in blocking solution (PBS/0.1% Triton X-100 and 4% normal goat serum /4% bovine serum albumin) for 1 h at room temperature (RT), with the primary antibodies for 48 h at 4°C and with a biotinylated secondary antibody (1:250; Vector Laboratories, Burlingame, CA, USA) for 2 h at RT. Immunoreactivity was visualized using the ABC amplification system (Vectastain ABC kit, Vector Laboratories, Burlingame, CA, USA) with 3,3'-diaminobenzidine tetrahydrochloride (DAB Metal Concentrate, Biogenex, Fremont, CA, USA) as substrate. After dehydration in graded ethanol and toluene solutions, the slides were coverslipped. Double staining for ataxin-7 and other proteins was performed as above using different combinations of primary antibodies and secondary antibodies coupled to fluorophores (1:500; Molecular Probes, Eugene, OR, USA). For immunofluorescence, nuclei were counterstained with DAPI (Sigma-Aldrich St Louis, MI, USA).

**Quantification of the microglial and astrocytic immunoreactivity**

GFAP and Iba1 immunoreactivity were quantified using Image J (NIH, Bethesda, USA). Laserpower, numeric gain and magnification were kept constant between animals to avoid potential technical artefacts. Images were first converted to 8-bit gray scale and binary thresholded to highlight a positive staining. Five sections per mouse were quantified and the average value was calculated. Mean immunoreactivity intensity values were measured for either Iba1 or GFAP immunoreactivity. Analysis of data was blind with respect to treatments.
